# Triphenylamine‐Functionalized Metal Nanoclusters for Efficient and Stable Perovskite Solar Cells

**DOI:** 10.1002/advs.202410796

**Published:** 2024-11-05

**Authors:** Lin Wang, Jieru Du, Jiahao Wu, Zi‐Ang Nan, Simin Li, Xiongkai Tang, Zhenlang Xie, Qinghua Xu, Xuekun Gong, Jinlu He, Ruihao Chen, Nanfeng Zheng, Hui Shen

**Affiliations:** ^1^ College of Energy Materials and Chemistry Inner Mongolia University Hohhot 010021 China; ^2^ College of Chemistry and Chemical Engineering Inner Mongolia University Hohhot 010021 China; ^3^ State Key Laboratory of Solidification Processing School of Materials Science and Engineering Northwestern Polytechnical University Xi'an 710072 China; ^4^ CAS Key Laboratory of Design and Assembly of Functional Nanostructures and Fujian Provincial Key Laboratory of Nanomaterials Fujian Institute of Research on the Structure of Matter Chinese Academy of Sciences Fuzhou 350002 China; ^5^ New Cornerstone Science Laboratory State Key Laboratory for Physical Chemistry of Solid Surfaces Collaborative Innovation Center of Chemistry for Energy Materials and National & Local Joint Engineering Research Center of Preparation Technology of Nanomaterials College of Chemistry and Chemical Engineering Xiamen University Xiamen 361005 China; ^6^ College of Food Science and Engineering Guangdong Ocean University Yangjiang 529500 China; ^7^ Innovation Laboratory for Sciences and Technologies of Energy Materials of Fujian Province (IKKEM) Xiamen 361102 China

**Keywords:** Alkyne ligands, cluster compounds, crystal structure, organic–inorganic hybrid materials, solar cells

## Abstract

Reported herein is a ligand engineering strategy to develop photoelectric active metal nanoclusters (NCs) with atomic precision. Triphenylamine (TPA), a typical organic molecule in the photoelectric field, is introduced for the first time to prepare atomically precise metal NCs that prove effective in the fabrication of perovskite solar cells (PSCs). The scalable synthetic prototype, unique electronic strucuture, and atomically precise structure of the cluster ([(AgCu)_37_(PPh_3_)_8_(TPA‐C≡C)_24_]^5+^) are illustrated in this work. When being employed as a buffer layer in the perovskite/HTL interface of PSCs, significantly enhanced performance is observed. The resultant n‐i‐p devices achieved a substantial power conversion efficiency as high as 25.1% and long‐term stability. The findings offer valuable insights into preparing functionalized metal NCs that play multiple roles in improving the performance of the device: while the inorganic metal core enhances conductivity, the organic TPA shell promotes the “carrier transfer” between the perovskite and HTL layer and prevents the perovskite from corrosion.

## Graphical abstract

Here it is reported that triphenylamine‐functionalized metal NCs represent a new class of inorganic–organic hybrid materials for PSCs. The resulting solar cell achieves a PCE of 25.1% and long‐term stability. While the inorganic metal component strengthens the conductivity, the organic shell optimizes the carrier transfer between perovskite and HTL.

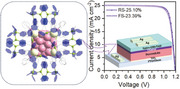

## 1 Introduction

Organic ligand‐protected metal nanoclusters (OLPMNCs), which consist of both an organic shell and an aggregation of metal atoms, are emerging as a novel class of organic–inorganic hybrid materials.^[^ ^1^ ^]^ The merits of OLPMNCs, including absolute homogeneity in size, composition, and charge, well‐defined geometric structures, and ready functionalization with single‐atom precision, endow them great potency in applications.^[^ ^2^ ^]^ To date, OLPMNCs have found utility in various fields such as catalysis, optics, electronics, biology, and energy utilization (such as photovoltaics).^[^ ^3^ ^]^ Recent studies have focused on enhancing the performance of OLPMNCs through the introduction of functionalized surface ligands, which is inspiring and steering our focus on the design of functionalized OLPMNCs to enhance their performance in specific target areas.^[^ ^4^ ^]^

Regular n‐i‐p perovskite solar cells (PSCs) are receiving intense attention due to low cost, solution processability and high efficiency.^[^ ^5^ ^]^ They are typically fabricated by the sandwich of organic‐inorganic metal halide perovskites between an electron‐transport layer (e.g., TiO_2_) and a hole‐transport layer (HTL, commonly 2,2′,7,7′‐tetrakis(N,N‐di‐p‐methoxyphenyl)‐amine‐9,9′‐spirobifluorene, Spiro‐MeOTAD) with front transparent conductive metal oxide and back metal electrodes.^[^ ^6^ ^]^ Over the past decades, various strategies have been investigated to enhance the power conversion efficiency (PCE) and stability of PSCs, among which optimizing the perovskite/HTL interface proves efficacious.^[^ ^7^ ^]^ Versatile materials, including organic (small molecules), inorganic (nanoparticles), and organic‐inorganic hybrid (self‐assembled monolayers) are promising in tailoring the interface of perovskite/HTL.^[^ ^8^ ^]^ While inorganic materials feature superior carrier mobility and conductivity, organic ones are more effective in modulating the structure, electronic structure, and functionalization of the interface.^[^ ^9^ ^]^

Notably, several embryonic studies have highlighted the potency of ligand‐stabilized metal NCs as hybrid inorganic‐organic materials for modifying the interfaces of PSCs. For example, Chen et al. added Au clusters with 3‐methylbenzenethiol in perovskite precusor to improve the light absorption ability of perovskite and passivate the defects with PCE of 20.6%.^[^ ^10^ ^]^ To further improve the efficiency, Wang et al. introduced hydrophobic fluorinated Au cluster as an interface modifier to increase the carrier transfer and extraction, and the fabricated formamidinium lead iodide (FAPbI_3_) PSCs achieved PCE of 24.02% with excellent stability.^[^ ^11^ ^]^ Recently, a bovine serum albumin‐functionalized Au NC which interacts strongly with the electron transport layer was used to reconstruct the bottom interface for high‐quality crystallization and improve carrier transfer, and the FAPbI_3_ based device attained a power conversion efficiency of 25.0%.^[^ ^12^ ^]^ However, the lack of well‐defined structures of employed clusters or low PCEs of the resulting PSCs significantly impedes the establishment of structure‐property relationships.^[^ ^12^, ^13^ ^]^

Our group has a strong interest in the exploration of OLPMNC‐based photoelectric materials. Close examination of the literature has showcased that triarylamine derivatives represent a crucial class of materials in photovoltaics.^[^ ^14^ ^]^ Triphenylamine motif‐based molecules are always excellent hole transport layer materials in photovolatics, such as poly (triarylamine) (PTAA) and Spiro‐MeOTAD.^[^ ^15^ ^]^ Especially, these derivatives exhibit strong compatibility with the spiro layer, thereby facilitating hole transfer or hole shuttling from the perovskite layer to the hole transport layer (HTL) in PSCs.^[^ ^16^ ^]^

Herein, we thus designed and synthesized triphenylamine (TPA)‐functionalized metal NCs of atomic precision and applied them as the buffer layer of perovskite/HTL in PSCs. Our studies reveal that the TPA‐decorated NCs could significantly promote the PCE and stability of PSC devices, much superior to those modified by either unfunctionalized NCs or sole ligand molecules. The improved performance stems from the synthetic effect of the clusters: while the organic ligand component promotes the “carrier transfer”, the inorganic metal part enhances the conductivity.

## 2 Results and Discussion

**Figure 1:**
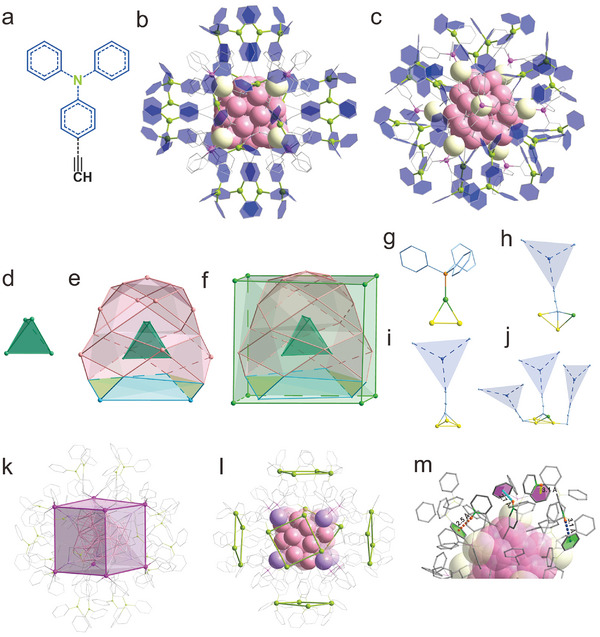
X‐ray single crystal diffraction structure of the (AgCu)_37_ cluster. a) Molecular structure of the TPA‐C≡CH ligand. b,c) Total structure of (AgCu)_37_ in the front and side view, respectively. Color legends: rose, Ag/Cu; light yellow, Ag; lime, N; pink, P, gray, C. d–f) The shell‐by‐shell anatomy of the cluster: the inner M_4_ tetrahedron (d), the M_25_ polyhedron in the second layer (e), and the outermost Ag_8_ cube (f). g–j) Coordination modes of surface ligands to the metal framework. Color legends: rose, green, gold, pink, pale blue, Ag/Cu; sea green, Ag; lime, N; orange, P. k) The 8 TPP ligands collectively form a cube with a side length of 9.6 Å. l) The 24 TPA‐C≡C ligands are systematically distributed around the six faces of the Ag_8_ cube. m) The cluster exhibits four distinct types of intracluster C─H···π interactions.

TPA‐functionalized metal clusters were prepared by a two‐step synthesis protocol (see Supporting Information for complete procedural details). To incorporate the photovoltaic TPA group into the metal NCs structure, an alkyne ligand containing TPA (referred to as TPA‐C≡CH, as illustrated in **Figure** **1**a) was initially designed. This ligand was ready to react with AgNO_3_ under basic conditions to afford the yellow complex of TPA‐C≡CAg. The synthesis of the target clusters was achieved through the reduction of TPA‐C≡CAg using the reducing agent bis(triphenylphosphine)copper(I) borohydride ((PPh_3_)_2_CuBH_4_) in a one‐pot reaction.^[^ ^17^ ^]^ As displayed in Figure S1 (Supporting Information), the UV–vis spectrum of the raw product showcase distinct peaks at 307, 334, and 585 nm, respectively, which is a strong indication that metal NCs are formed. The final crystalline product was obtained by introducing *n*‐hexane into the mixed solution. Notably, this facile synthetic process is capable of a gram‐scale in NCs’ preparation, which is significant for application investigation (Figure S2, Supporting Information).

The analysis of the crystalline product through X‐ray single crystal diffraction presents the creation of TPA‐functionalized metal clusters of [(AgCu)_37_(PPh_3_)_8_(TPA‐C≡C)_24_]^5+^ (Figures S3–S5, Supporting Information, labeled as (AgCu)_37_ hereafter). As one can see, the overall structure of (AgCu)_37_ adopts a cubic configuration, where 8 Ag atoms serve as the vertices of the cube, and the remaining 29 atoms are included in an ordered manner (Figure 1b,c). In the crystal structure, all phosphine ligands are attached to the Ag vertexes of the Ag_8_ cube while alkynyl ligands are anisotropically distributed around the cube. The total length as determined from the crystal structure is ≈2.85 nm (Figure S6, Supporting Information), which closely aligns with the 2.56 nm observed in transmission electron microscopy (Figure S7, Supporting Information).

Structural dissection shows that the metal core of the (AgCu)_37_ cluster is composed of a M_4_ (M = Ag or Cu) tetrahedron with an average bond length of 2.75 Å (Figure 1d). Surrounding this tetrahedron is a 25‐atom cage comprising 11 irregular quadrilaterals (highlighted in rose and pale blue), and 2 triangles (highlighted in yellow), respectively (Figure 1e). The outermost layer of the cluster contains 8 Ag atoms arranged in a cubic shape with a side length of 6.94 Å (Figure 1f). Additionally, all PPh_3_ ligands in the cluster terminate the structure in a μ_1_ manner (Figure 1g), while the alkynyl ligands exhibit 5 coordination modes: μ_3_ (Ag, M, M), μ_3_ (M, M, M), μ_4_ (Ag, M, M, M), μ_2_ (M, M), and μ_1_ (M), respectively (Figure 1h–j). Each of the eight TPP ligands is strongly attached to an Ag atom at the apex of the metal core (average Ag‐P bond length of 2.340 Å), themsevels presenting a P_8_ cube with a side length of 9.6 Å (Figure 1k). Intriguingly, the 24 TPA‐C≡C ligands are also meticulously arranged across the six faces of the Ag_8_ cube. Specifically, every four TPA‐C≡C ligands form a square with a side length of 6.3 Å, and these six squares encase the metal framework in a staggered manner (Figure 1l). The M‐C bond lengths fall within the range of 1.230‐2.760 Å. Notably, abundant intracluster C─H···π interactions are present on the surface of the cluster (depicted in Figure 1m). These interactions can be categorized into four types based on the groups involved: C─H (Ph‐C≡C)···π (TPP) at 2.7 Å, C─H (C≡C‐Ph)···π (TPP) at 3.1 Å, C─H (Ph‐C≡C)···π (Ph‐C≡C) at 2.5 Å, and C─H (C≡C‐Ph)···π (C≡C‐Ph) at 3.1 Å, respectively, where Ph‐C≡C denotes the phenyl groups of TPA connecting to the C≡C and C≡C‐Ph denotes the those of TPA in the periphery.

**Figure 2:**
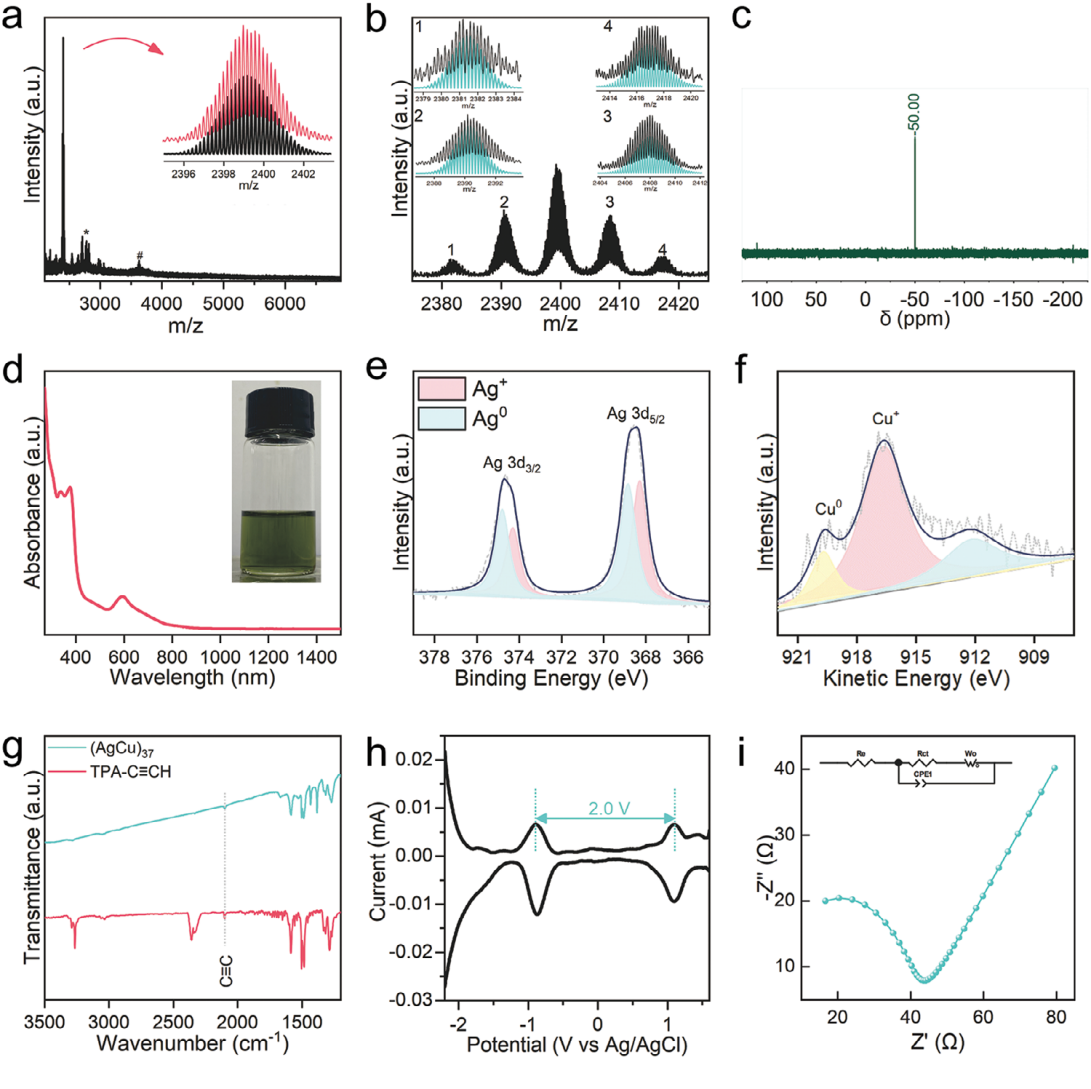
Characterization of the (AgCu)_37_ cluster. a) Total ESI‐MS spectra of the cluster in the positive mode. Inset is the comparison of the measured (red trace) and simulated (black trace) isotopic patterns of the molecular ion peak of [Ag_25_Cu_12_(PPh_3_)_8_(TPA‐C≡C)_24_]^5+^. b) The enlarged HRESI‐MS spectra and the comparison between the measured (black trace) and simulated (green trace) isotopic patterns of other ion peaks. (c) ^31^P NMR spectra of (AgCu)_37_ in d_6_‐DMSO. d) UV–vis–NIR spectra of (AgCu)_37_. Inset is the photograph in the solution form. e,f) XPS of Ag 3d (e) and Cu LMM (f) of the cluster. g) FT‐IR spectra of (AgCu)_37_ (green line) and TPA‐C≡CH ligand (red line). h) The HOMO‐LUMO gap of the (AgCu)_37_ cluster calculated from its DPV spectrum. i) EIS of (AgCu)_37_.

Electrospray ionization mass spectrometry (ESI‐MS) represents a crucial analytical technique utilized for determining the composition and charge of cluster compounds. A group of peaks centered at ≈2400 m z^−1^ are observed in the ESI‐MS spectrum of (AgCu)_37_ in a positive mode (**Figure** **2**a,b). We confirmed that the most prominent peak corresponds to [Ag_25_Cu_12_(PPh_3_)_8_(TPA‐C≡C)_24_]^5+^, with its experimentally derived isotopic pattern closely matching with the simulated pattern (inset of Figure 2a). Additional peaks arise due to the mutual exchange of Ag and Cu within the metal framework, reflecting the site‐occupancy disorder identified in crystallographic analysis. Noteworthy aspects of the pristine cluster's formula are that: 1. It possesses a charge high up to “+5”, which is quite uncommon in cluster compounds. The ability to accommodate significant positive charges within the cluster structure may be attributed to the presence of TPA units on its surface. These units facilitate redox reactions, enabling the transition between the neutral and cationic forms of the nitrogen atoms.^[^ ^18^ ^]^ 2. Eight free valence electrons are present in the cluster, indicating it is probably an eight‐electron superatom.^[^ ^19^ ^]^ The behavior of the cluster in solution has also been conducted by nuclear magnetic resonance (NMR). The ^1^H NMR spectrum of (AgCu)_37_ in d_6_‐DMSO displays peaks in a range of 7.42–7.63 ppm which are associated with phenyl groups in the cluster (Figure S8, Supporting Information). The ^31^P NMR spectrum shows a single and sharp peak, verifying a consistent chemical environment for the phosphine ligands, at least on the timescale of NMR measurement (Figure 2c). In dichloromethane, the cluster appears pale green, exhibiting characteristic peaks corresponding to 334, 372, and 595 nm, respectively in its UV–vis spectrum (Figure 2d).

X‐ray photoelectron spectroscopy (XPS) studies of the (AgCu)_37_ cluster shows the binding energies of Ag 3d_5/2_ at 368.9 and 368.3 eV and Ag 3d_3/2_ at 374.8 and 374.3 eV (Figure 2e), respectively, indicating that both reduced and oxidized Ag atoms are present in the cluster. Similarly, copper atoms in the cluster, as evidenced by Cu LMM spectroscopy (919.2 and 916.7 eV), exhibit both metallic and oxidation states (Figure 2f). The Fourier transform infrared (FT‐IR) spectra of (AgCu)_37_ show a C≡C peak at 2100 cm^−1^. The similar value to that of TPA‐C≡CH ligand strongly suggests for the presence of alkyne ligand in the cluster (Figure 2g). In addition, a homogeneous distribution of the constituent elements (Ag, Cu, P, N, and C) is observed in dispersive X‐ray spectrometry images of the cluster (Figure S9, Supporting Information). The powder X‐ray diffraction analysis of the clusters has verified the purity of the initial sample (Figure S10, Supporting Information). The time‐dependent UV‐vis spectra of the cluster in solution under ambient conditions, as depicted in Figure S11 (Supporting Information), declare its moderate stability. The HOMO‐LUMO bandgap of the (AgCu)_37_ clusters which was determined through differential pulse voltammetry (DPV) showed a bandgap of 2.0 V, underscoring the significant electronic stability of the clusters (Figure 2h). Moreover, distinct from most organic compounds that are insulators (*σ* < 10^−9^ S cm^−1^), the cluster demonstrates a notable conductivity of 2.5 × 10^−4^ S cm^−1^, as calculated from the results of electrochemical impedance spectroscopy (EIS) tests (Figure 2i).

**Figure 3:**
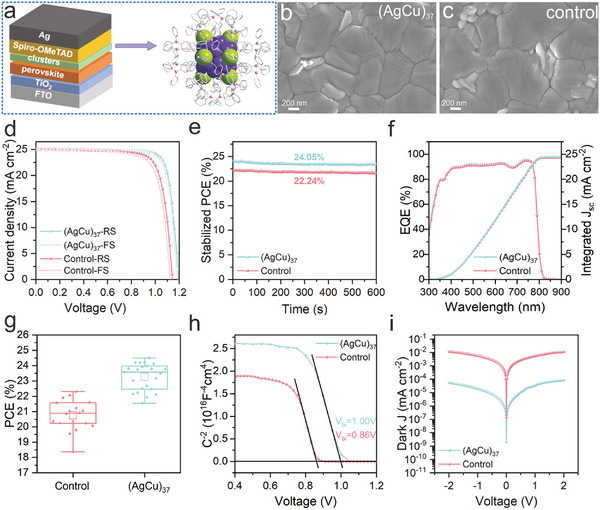
(AgCu)_37_ cluster for PSCs. a) Schematic of PSC structure with (AgCu)_37_ nanoclusters as interface layer. b,c) Top‐view SEM images of (AgCu)_37_‐moidified (b) and control (c) perovskite films. d) *J–V* curves and (e) Stabilized PCEs of the champion PSCs with (AgCu)_37_‐moidified and control perovskite films. f) EQE spectra and integrated *J*_sc_ of the champion PSCs with (AgCu)_37_‐moidified and control perovskite films. g) PCE of Distribution of 20 individual PSCs based on the (AgCu)_37_‐modified and control perovskite films. h) Mott−Schottky analysis of PSCs with (AgCu)_37_‐moidified and control perovskite films. i) *J–V* curves of PSCs with (AgCu)_37_‐moidified and control perovskite films under dark conditions.

Featuring semiconductor nature, presence of TPA units that facilitate the hole transfer from perovskite into the HTL layer, and high conductivity, it occurs to us that the cluster is promising in the modification of the perovskite/HTL interface in PSCs. To evaluate the impact of the (AgCu)_37_ NC in the n‐i‐p PSCs, we fabricated devices following the FTO/TiO_2_/perovskite/cluster/spiro‐OMeTAD/Ag (**Figure** **3**a). The cluster modification was performed by a spin‐coating process utilizing Cs_0.05_FA_0.85_MA_0.1_PbI_2.9_Br_0.1_ as the perovskite film, and isopropanol and acetonitrile as solvents. As shown in Figure 3b,c, the dot‐like (AgCu)_37_ cluster was evenly dispersed onto the surface of the perovskite film, maintaining the perovskite grain morphology. EDS mapping images confirmed the presence of (AgCu)_37_ cluster (Figure S12, Supporting Information). FT‐IR presented the shift of characteristic stretching vibration peaks of C─N, indicating significant interaction between TPA and PbI_2_ (Figure S13, Supporting Information). In the steady PL and TRPL spectra, the longer average decay time (τ) of 176 ns in the (AgCu)_37_‐modified perovskite film than that of 126 ns in the control film indicated that the (AgCu)_37_ cluster reduced the interfacial trap density (Figure S14, Supporting Information). Impressively, the (AgCu)_37_‐modified device exhibited remarkably higher power conversion efficiencies (PCEs) of 24.5% and 23.88% during reverse (RS) and forward (FS) scans, respectively, much surpassing those (RS 22.31%, FS 21.27%) of the control devices (without the addition of clusters, Figure 3d and **Table** **1**). Furthermore, the (AgCu)_37_‐modified device exhibited less hysteresis and an improving stabilized PCE (24.05%) compared to the control devices (22.24%) under maximum power tracking for 600 s (Figure 3e).

**Table 1:** Photovoltaic parameters of control and (AgCu)_37_‐modified PSCs.

Entry	*V_oc_* [V]	*J*_sc_ [mA cm^−2^]	FF [%]	PCE [%]
Control	1.143	25.06	77.87	22.31
(AgCu)_37 –_ PSCs	1.192	25.13	81.78	24.50
(AgCu)_37 –_ FAPbI_3_ PSCs	1.204	25.48	81.86	25.10

The results depicted in Figure 3f show that the integrated short circuit density *(J*_sc_) values of the (AgCu)_37_‐modified and control devices were quite similar measuring at 24.47 and 24.32 mA cm^−2^, respectively, which matches with the *J*_sc_ values of the *J–V* curves. We further made a statistical analysis of the photovoltaic parameters based on 20 individual (AgCu)_37_‐modified and control PSCs that confirmed the (AgCu)_37_ modification improved reproducibility, as evidenced in Figure 3g and Figure S15 (Supporting Information). In this case, the *V*_bi_ is from 0.86 to 1.00 V after modification (Figure 3h). That said, new advances in (AgCu)_37_‐modified devices have allowed significantly decreasing dark current (Figure 3i) as well as a larger recombination resistance in Nyquist plots (Figure S16, Supporting Information). The space‐charge‐limited current (SCLC) technique presented a reduction of defect density (Figure S17, Supporting Information). Above results demonstrated, probably due to the formation of π‐π conjugation between the TPA ligands of the cluster and the TPA groups of the spiro‐OMeTAD, that (AgCu)_37_ modification could effectively promoted the transfer and extraction of holes from the perovskite layer to the HTL layer. In the meanwhile, the (AgCu)_37_ metal core contributed to increased conductivity, thereby synergistically improving the PSC performance. And, from ultraviolet photoelectron spectroscopy (UPS) measurements, the obtained lower valence band energy (E_VB_) of −5.21 eV for the (AgCu)_37_ film in comparison with −5.12 eV for the control film (Figure S18, Supporting Information) indicates the better hole transfer in the perovskite/spiro‐OMeTAD interface with the more matched energy level by (AgCu)_37_ modification (Figure S19, Supporting Information).

**Figure 4:**
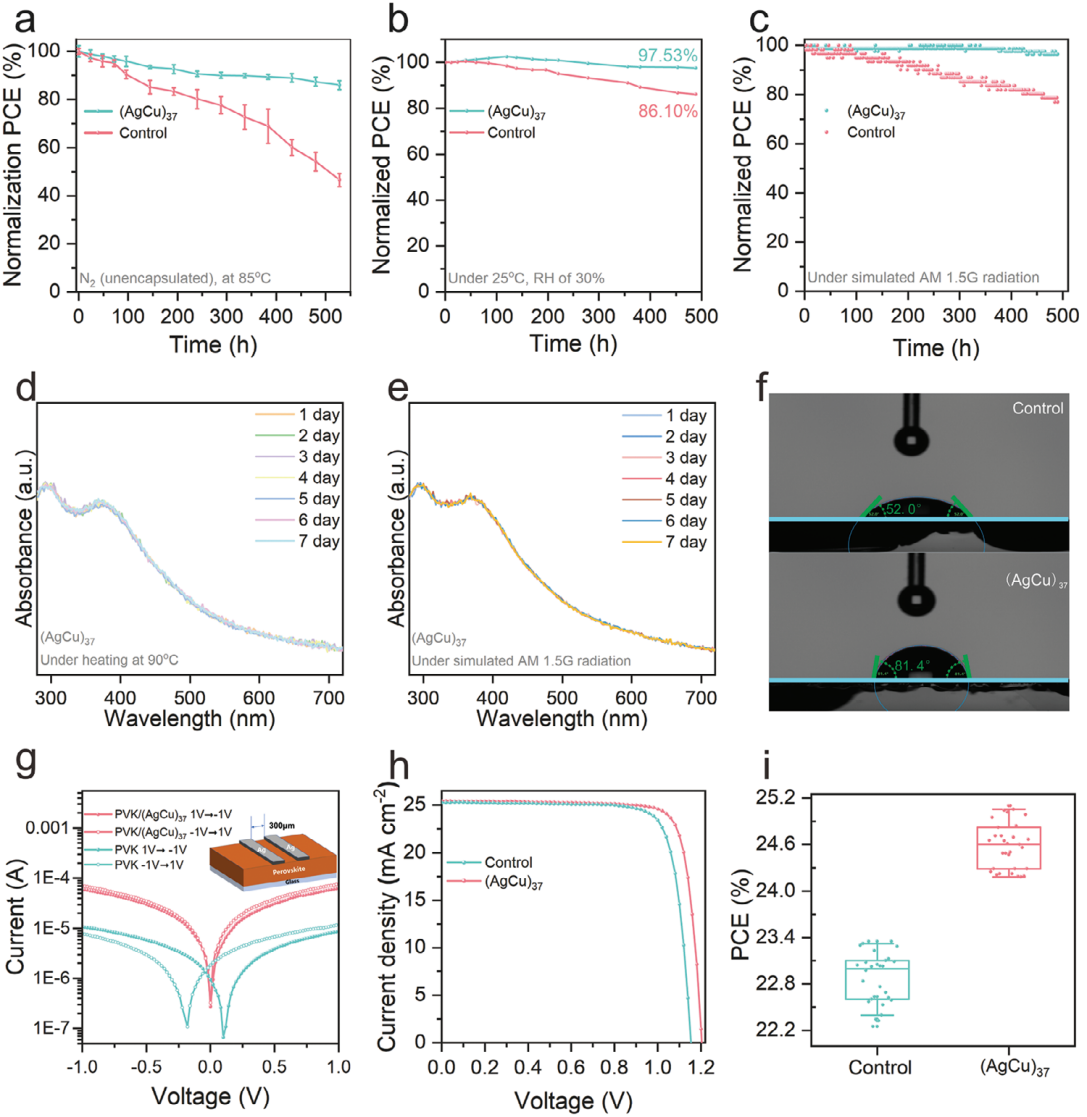
Stability evaluation and performance optimization of (AgCu)_37_‐modified PSCs. a–c) Moisture (a), thermal (b), and operational stability (c) of (AgCu)_37_‐modified PSCs. d,e) Time‐dependent UV–vis diffuse reflectance spectra of the (AgCu)_37_ cluster under thermal (d) and photo (e) treatment. f and g) The comparison of contact angle (g) and logarithmic *J–V* curves between the control and (AgCu)_37_‐modified perovskite film. h and i) The *J–V* curves (h) and PCEs (i) of the control and (AgCu)_37_‐modified FAPbI_3_ PSC films.

We then assessed the stability of fabricated PSCs under various conditions. First, we sought to test the thermal stability of PSCs, in which unencapsulated devices were subject to N_2_ atmosphere at 85 °C. As shown in **Figure** **4**a, the (AgCu)_37_‐modified device retained 85.97% of the initial PCE over 500 h while the control PSCs remained at only 46.56% during the same time. Next, the moisture stability of the PSCs under storage conditions (25 °C, relative humidity of 30%) was evaluated. As illustrated in Figure 4b, the (AgCu)_37_‐modified device retained 97.53% of the initial PCE, while the control PSC degraded to 86.1% of the original efficiency after 488 h. To further evaluate operational stability of the device, we carried out light stability assessment, in which unencapsulated devices were held at the maximum power point (MPP) under simulated AM 1.5G radiation in N_2_. The (AgCu)_37_‐modified PSCs retained 96.5% of the original PCE after 490 h, much higher than that (77.05%) of the controlled one (Figure 4c).

The heightened stability of the (AgCu)_37_‐modified device is a result of multiple attributes of the triphenylamine‐functionalized metal nanoclusters. First, the cluster is highly robust. As shown in Figure 4d,e, UV–vis diffuse reflectance spectra of the (AgCu)_37_ cluster maintained its profiles for at least 7 days, either under heating at 90 °C (Figure 4d) or at AM 1.5G illumination (Figure 4e). Such a robust structure could firmly anchor the metal core and insulate the contact between metallic atoms and perovskite, restraining the general irreversible degradation upon the metal diffusion and migration. Second, the surface functionalization of the metal clusters with TPA ligands make the surface of the perovskite film highly hydrophobic. In comparison to the contact angle of the control perovskite film (52.0°), the (AgCu)_37_‐modified film showed a larger contact angle of 81.4° (Figure 4f). The high hydrophobicity of the benzene ring structure in the cluster could effectively prevent the penetration of moisture and rationalize moisture stability of the (AgCu)_37_‐modified device. Third, the TPA ligands could form hydrogen bonding with perovskite, which suppresses the ionic migration and favors for thermal and operational stability. As shown in the logarithmic *J–V* curves (Figure 4g), the control films were shifted due to the difference in scanning direction, whereas the forward and reverse scanning curves of the (AgCu)_37_‐modified films almost overlapped. Moreover, the characteristic peaks of N‐H of organic cations of perovskite (MAI and FAI) are shifted upon the mixing with TPA ligands (Figure S20, Supporting Information). These data strongly suggest the formation of hydrogen bonds between the TPA ligands of clusters and the organic cations of perovskite. Finally, the π‐π interaction between the TPA ligands of the cluster and the TPA groups of the spiro‐OMeTAD optimized buried interface without voids (Figure S17, Supporting Information), further enhancing the stability of the device. Collectively, the highly robust structure of (AgCu)_37_, the hydrophobic tails of TPA ligands, the multiple bonding (hydrogen and π‐π) formed between TPA groups and other components (perovskite and spiro‐OMeTAD) contribute the enhancement of thermal, moisture and operational stability of (AgCu)_37_‐modified device.

To accurately elucidate the enhanced performance of (AgCu)_37_ modification and ascertain the importance of TPA‐functionalized surface and metal core in PSCs, other related clusters ([Ag_13‐x_Cu_6+x_(^t^BuC_6_H_4_C≡C)_14_(PPh_3_)_6_](SbF_6_)_3_, referred to as Ag_13‐x_Cu_6+x_ and [Ag_25_Cu_4_(PhC≡C)_12_(PPh_3_)_12_Cl_6_H_8_](SbF_6_)_3_, referred to as Ag_25_Cu_4_) and TPA‐C≡CH ligand were fabricated as reference to modify the interface of perovskite/HTL (Figures S21–S23, Supporting Information).^[^ ^17^, ^20^ ^]^ As depicted in Figures S24 and S25 (Supporting Information), while Ag_13‐x_Cu_6+x_ (RS 23.07%) marginally improved the PCEs, Ag_25_Cu_4_ (RS 22.32%) did not exhibit enhancement. Moreover, attributing to the TPA molecular passivation effect, a slight improvement was observed in the PSC modified by the TPA‐C≡CH ligand. The conductivities of the clusters and clusters with spiro‐OMeTAD were subsequently tested and analyzed (Figure S26, Supporting Information), revealing the following order: (AgCu)_37_ > Ag_13x_Cu_6+x_ > Ag_25_Cu_4_. We are well acquainted with the fact that the TPA‐functionalized metal NCs are ideal candidates for the interface modification of PSCs, which is beneficial to both enhanced conductivity and passivation effect.

The above studies demonstrate that the (AgCu)_37_ cluster as a buffer layer in the perovskite/HTL interface is beneficial to enhance the performance of Cs_0.05_FA_0.85_MA_0.1_PbI_2.9_Br_0.1_ PSCs. We thus verify the universality of the cluster modification and further improve the device efficiency. Experimentally, when (AgCu)_37_ was used to modify the FAPbI_3_ perovskite film, the fabricated device turned out to be also a high‐performance solar cell. As shown in Figure 4h, open‐circuit voltage (*V*_OC_) and *J*_SC_ of (AgCu)_37_‐modified FAPbI_3_ PSCs were enhanced by 0.05 V and 0.2 mA cm^−2^, respectively. The PCE was even increased from 23.39% to 25.10%, a champion value in cluster‐modified perovskite systems (Figure 4i; Figure S27; Tables S1 and S2, Supporting Information).

## 3 Conclusion

In conclusion, we have a deep bench of TPA‐functionalized metal NCs that represents a novel class of nanoscale‐sized, and highly efficient photoelectric materials at the atomic level. Taking the novel cluster of [(AgCu)_37_(PPh_3_)_8_(TPA‐C≡C)_24_]^5+^ that has been fully characterized and structurally determined as a prototype, we find that it can obviously improve the performance of PSCs when being applied to modify the perovskite/HTL interface. The resultant PSC devices dramatically elevate a power conversion efficiency to 25.10% and a long‐term stability, superior to those modified by unfunctionalized clusters and sole organic ligands. The enhanced performance of the PSCs stems from the synesthetic effect of the TPA‐functionalized metal clusters. While the inorganic metal component strengthens the conductivity that is often missing in organic modifiers, the functionalized organic shell on the other hand optimizes the “carrier transfer” between perovskite and HTL. It is anticipated that atomically precise metal NCs with functionalities will attract increasing interest in the future.^[^ ^17^, ^20^, ^21^ ^]^

## Conflict of Interest

The authors declare no conflict of interest.

## Supporting information



Supporting Information

## Data Availability

The data that support the findings of this study are available from the corresponding author upon reasonable request.
