# Supplementary material for: Triphenylamine‐Functionalized Metal Nanoclusters for Efficient and Stable Perovskite Solar Cells
Source: Adv Sci (Weinh). 2024 Nov 5;12(1):2410796. doi: 10.1002/advs.202410796 (PMC11714199; doi:10.1002/advs.202410796)
Supplement: Supplementary file 1 — Supporting Information [file ADVS-12-2410796-s001.docx]

Supporting Information

**Triphenylamine-Functionalized Metal Nanoclusters for Efficient and Stable Perovskite Solar Cells**

*Lin Wang,^[a][b]#^ Jieru Du,^[c]#^ Jiahao Wu,^[c]#^ Zi-Ang Nan,^[d]^ Simin Li,^[a]^ Xiongkai Tang,^[e]^ Zhenlang Xie,^[f]^ Qinghua Xu,^[a]^ Xuekun Gong,^[a]^ Jinlu He,^[b]^ Ruihao Chen,*^[c]^ Nanfeng Zheng,*^[e][g]^ Hui Shen*^[a]^*

a. College of Energy Materials and Chemistry, Inner Mongolia University, Hohhot 010021, China

b. College of Chemistry and Chemical Engineering, Inner Mongolia University, Hohhot 010021, China

c. State Key Laboratory of Solidification Processing, School of Materials Science and Engineering, Northwestern Polytechnical University, Xi’an 710072, China

d. CAS Key Laboratory of Design and Assembly of Functional Nanostructures, and Fujian Provincial Key Laboratory of Nanomaterials, Fujian Institute of Research on the Structure of Matter, Chinese Academy of Sciences, Fuzhou 350002, China

e. New Cornerstone Science Laboratory, State Key Laboratory for Physical Chemistry of Solid Surfaces, Collaborative Innovation Center of Chemistry for Energy Materials, and National & Local Joint Engineering Research Center of Preparation Technology of Nanomaterials, College of Chemistry and Chemical Engineering, Xiamen University, Xiamen 361005, China

f. College of Food Science and Engineering, Guangdong Ocean University, Yangjiang 529500, China

g. Innovation Laboratory for Sciences and Technologies of Energy Materials of Fujian Province (IKKEM), Xiamen 361102, China

* Corresponding authors.

# These authors contributed equally to this work

E-mail: shen@imu.edu.cn, rhchen@nwpu.edu.cn, nfzheng@xmu.edu.cn

**Experimental Details**

**Materials.** 4-ethynyl-N,N-diphenylaniline (C_20_H_15_N, 97%), sodium hexafluoro antimonate (NaSbF_6_, 98%), and bis(triphenylphosphine)copper(I) borohydride [(PPh_3_)_2_CuBH_4_, 98%] were purchased from Bide (Shanghai, China). We obtained cesium iodide (CsI, 99.50%), methylamine hydrochloride (MACl, 99.50%), methylammonium bromide (MABr, 99.50%), formamidine Iodide (FAI, 99.50%) and lead iodide (PbI_2_, 99.99%), Spiro-OMeTAD (> 99.80%), Li-TFSI (> 99.90%), Co-TFSI (> 99.90%) and tBP (> 99.90%) from p-OLED. Silver nitrate (AgNO_3_, 99.8%) was purchased from Aladdin (Shanghai, China). Triethylamine [(C_2_H_5_)_3_N, 99.5%] was purchased from Adamas beta (Shanghai, China). Dichloromethane (CH_2_Cl_2_, A.R.), methanol (CH_3_OH, A.R.), ethanol (C_2_H_5_OH, A.R.), ethyl ether (C_4_H_10_O, A.R.), n-hexane (C_6_H_14_, A.R.) and acetonitrile (CH_3_CN, A.R.) were purchased from Sinopharm Chemical Reagent Co. Ltd. (Shanghai, China). Chlorobenzene (CB, 99.8%), dimethyl sulfoxide (DMSO, 99.9%), *N,N*-dimethylformamide (DMF, 99.8%), ethyl acetate (EA, 99.80%), and Isopropanol (IPA, 99.80%) were purchased from Sigma-Aldrich. FTO substrates (14 Ω sq^-1^) were acquired from Advanced Election Technology Co., Ltd. All reagents were used as received without further purification. Water used in all experiments was ultrapure. Related precursors were prepared according to the literatures.^[1]^

**General synthetic process of TPA-C≡CAg.** The precursor triethylamine-ethynyl silver (TPA-C≡CAg) was synthesized under base conditions. Briefly, to the solution of 4.5 mmol 4-ethynyl-N,N-diphenylaniline in 50 mL of acetonitrile, 0.5 mmol silver nitrate and 4.5 mmol triethylamine were added. The mixture was centrifuged after stirring for 3 h. The solid product was then washed with deionized water, ethanol, and ethyl ether sequentially, and dried under vacuum for 48 h.

**General synthetic process of [(AgCu)_37_(PPh_3_)_8_(TPA-C≡C)_24_]^5+^.** The nanostructured cluster was prepared by one-pot synthesis method. Typically, 9 mg of TPAC≡CAg (0.022 mmol) was completely dissolved in 1.5 mL of CH_2_Cl_2_ under ultrasonication. Then 5 mg sodium hexafluoroantimonate (0.02 mmol) in 0.5 mL of CH_3_OH was added and the mixture was stirred for 5 min. 6 mg of (PPh_3_)_2_CuBH_4_ (0.01 mmol) dissolved in 0.5 mL of DCM was added to above solution, and the supernatant was centrifuged after stirring for 1 h. The supernatant was subjected to the diffusion of n-hexane, and brown block crystals were obtained after one week. Yield: 3.86 mg, 35.9% based on Ag.

**X-ray Single Crystal Structure Analysis.** The single crystal diffraction data of (AgCu)_37_ clusters was collected using an Agilent Technologies SuperNova System X-ray diffractometer at 100 K. Data were collected at Cu Kα (λ= 1.54184 Å). After testing, processing was carried out using CrysAlis^Pro^. The structure was solved and refined using the full matrix least squares method based on F2 using ShelXT,^[2]^ ShelXL^[3]^ and Shelxle^[4]^ in Olex2.^[5]^ The thermal ellipsoid of the ORTEP diagram was completed with 50% probability. CCDC 2342657 contain the supplementary crystallographic data for this paper. Further details can be obtained from the cif files deposited at the Cambridge Crystallographic Data Centre and can be obtained free of charge on request. We note that the metal atoms in the cluster core is so disordered that the precise positioning is rather challenging. In this context, some of these positions in the metal core are potentially labeled incorrectly. But the composition and charge of the cluster has been confirmed by ESI-MS analysis

**Ultraviolet/visible/Near Infrared (UV-vis-NIR) spectra.** UV-vis-NIR spectra of (AgCu)_37_ cluster were collected on a PerkinElmer Lambda 1050+ Spectrophotometer at room temperature. The absorption spectra were measured by using quartz cuvette of 1 mm path length. The spectra were recorded in solution of dichloromethane or N,N-Dimethylformamide and the signal of the blank solvent was subtracted.

**UV-visible diffuse reflectance spectra.** The solid state UV-visible diffuse reflectance spectra of (AgCu)_37_ was recorded on Persee Tu-1590 UV-Vis spectrophotometer. BaSO_4_ was mixed with the (AgCu)_37_ cluster in prior to test. The signal of BaSO_4_ was subtracted during the measurement.

**Fourier transform Infrared (FT-IR) spectra.** The FT-IR spectra of (AgCu)_37_ clusters were collected with SHIMADZU IRAffinity-1S Spectrophotometer at room temperature.

**High-resolution electrospray ionization mass (HRESI-MS).** HRESI-MS data was recorded on an Agilent 6224 time-of-flight mass spectrometer in the positive mode. Firstly, the cluster sample was dissolved in ddichloromethane and filtered. The solution was then injected directly into the spectrometer via a syringe pump at a flow rate of 1.2 mL/h. The measurement parameters are as follows: capillary voltage: 4.0 kV; drying gas temp: 150 ºC; drying gas flow: 4 L/min; and nebulizer pressure: 20 psi.

**Nuclear magnetic resonance (NMR).** ^1^H and ^31^P NMR spectra of the cluster were recorded at room temperature on a Bruker AV-600 spectrometer with the with TMS and solvent residual signal as an internal reference. The NMR data were processed on MestReNova software.

**Powder X-ray diffraction (XRD).** X-ray diffraction patterns were obtained by an Empyrean X-ray diffractometer using nickel-filtered Cu Kα radiation at 40 kV and 40 mA.

**X-ray photoelectron spectroscopy (XPS).** Elemental Ag and Cu were determined by Thermo Fisher Scientific, ESCALABXI+ X-ray photoelectron spectroscopy at room temperature. The spectra were calibrated using the C 1s peak (284.5 eV).

**Dispersive X-ray spectrometry images (EDS).** EDS spectroscopy analysis was recorded on a Bruker XFlash6100 system.

**Electrochemical impedance spectroscopy (EIS).** Using an electrochemical workstation (CHI660E instrument), a standard three-electrode system (Ag/AgCl as the reference electrode, platinum wire as the counter electrode, and glassy carbon electrode as working electrode) and 0.5 M Na_2_SO_4_ solution as the electrolyte were used to conduct the photoelectrochemical activity test.

**Transmission electron microscopy (TEM).** TEM images were tested using JEM 1400.

**Photoluminescence**. The time-resolved photoluminescence (TRPL) and steady-state photoluminescence (PL) (excitation and 470 nm) spectra were obtained using a Pico Quant Fluo Time300 fluorescence spectrometer.

**EQE spectra.** The EQE spectra were measured by an Enli Technology EQE measurement system.

**Contact Angle Measurement.** The water contact angle of the film is measured using an optical contact angle meter (DSA 100, KRUSS), where a droplet of liquid is dropped on the surface of the film and stabilized to measure its angle with the film.

**Solar cell performance.** The *J-V* curve was tested using a solar simulator with Keithley 2400 source meter. Using Enli Technology solar simulator (IVS-KA5000), the light intensity was calibrated to AM1.5G/Sun (100 mW/cm^2^). The Logarithmic J-V curve was tested using a solar simulator with Keithley 2400 source meter. Tested by applying scanning voltages in different directions to the two terminal electrodes. The steady-state power out measurement of PSCs were measured using the Keithley 2420 source meter under 100 mW cm^-2^ (AM 1.5G illumination) with a bias voltage. For the moisture stability tests, unencapsulated PSCs were performed at 30% RH and room temperature environment.

**Device fabrication.** The laser etched FTO glass was ultrasonically cleaned with detergent, deionized water, IPA and ethanol respectively for 30 min. Then the cleaned FTO substrate was treated with UV ozone (UVO) for 15 min. And then, a 50 nm TiO_2_ layer was prepared by the CBD chemical water bath deposition method. The TiO_2_-coated substrate was treated with UVO for 15 minutes. For the perovskite film of Cs_0.05_FA_0.85_MA_0.1_PbI_2.9_Br_0.1_, the 1.55 M Cs_0.05_FA_0.85_MA_0.1_PbI_2.9_Br_0.1_ perovskite precursor solution was prepared by dissolving CsI (20.14 mg), MABr (17.36 mg), MACl (20.92 mg), FAI (226.58 mg), PbI_2_ (750.3 mg) and MACl (10.46 mg) in 1 mL of mixed solvents (V_DMF_:V_DMSO_ = 4:1). For the perovskite film of FAPbI_3_, 1.8 M FAPbI_3_ perovskite precursor solution was prepared by dissolving FAI (309.6 mg), PbI_2_ (829.8 mg) and MACl (27 mg) in 1 mL of mixed solvents (V_DMF_:V_DMSO_ = 4:1). To fabricate perovskite films, 25 μL perovskite solution was spin coated on TiO_2_ at 4000 rpm for 30 s, and 200 μL of EA anti-solvent was rapidly dripped on the perovskite films 10 s before the end, and then the film was heat-treated at 130 ^o^C for 10 min. For the cluster modification, 0.6 mg cluster (Ag_13-x_Cu_6+x_, (AgCu)_37_ or Ag_25_Cu_4_) or TPA-C≡CH molecule was dissolved in 1 mL of mixed solvents (V_IPA_:V_ACN_ = 6:4) to make the cluster solution, and then, 40 μL of the cluster solution was spin-coated onto perovskite films at 4000 rpm for 30 s without further anneal process. 72.3 mg Spiro-OMeTAD, 29 μL tBP, 32 μL Co-TFSI and 18 μL Li-TFSI were dissolved in 1 mL CB to make the HTL solution. Then, 20 μL of HTL solution was spin-coated onto perovskite/cluster substrates at 4000 rpm for 30 s. Ag or Au electrodes was subsequently deposited. The active area is 0.05 cm^2^ by a mask.


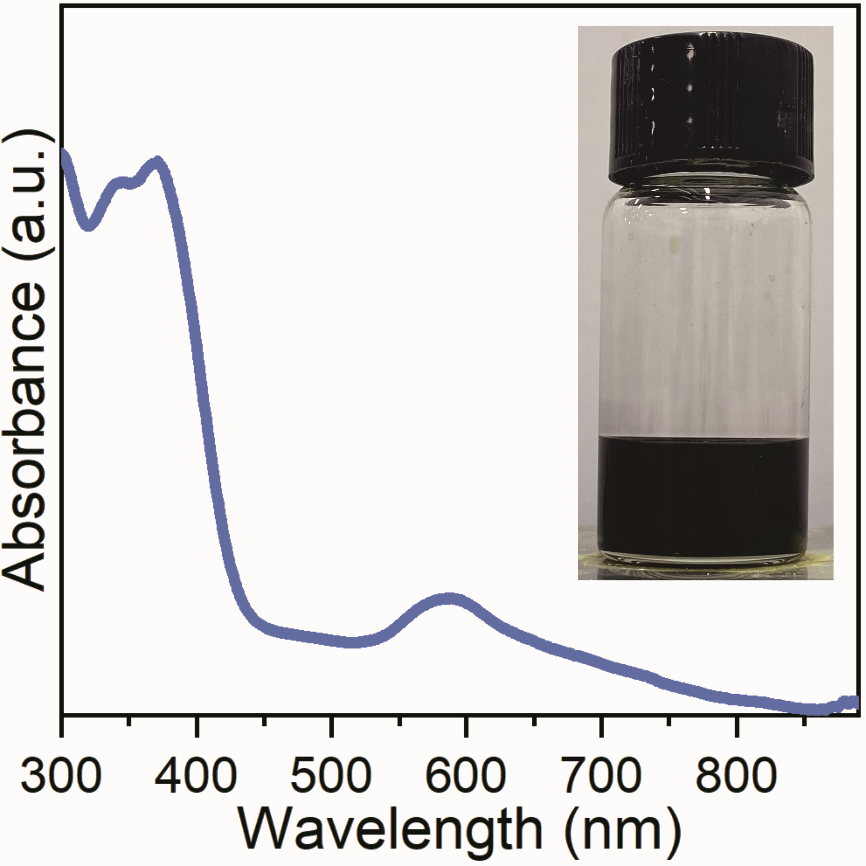


**Figure S1.** UV-vis spectrum and photographic images of raw product of (AgCu)_37_.


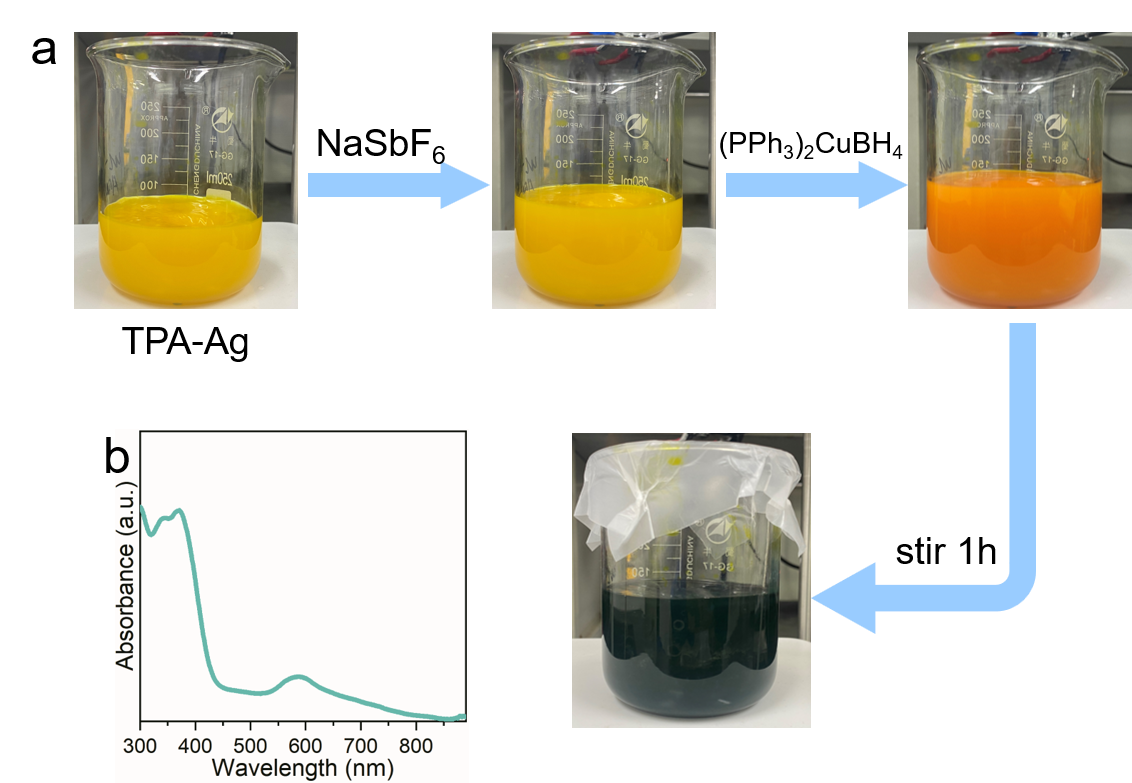


**Figure S2.** The procedure in the large-scale synthesis of (AgCu)_37_.


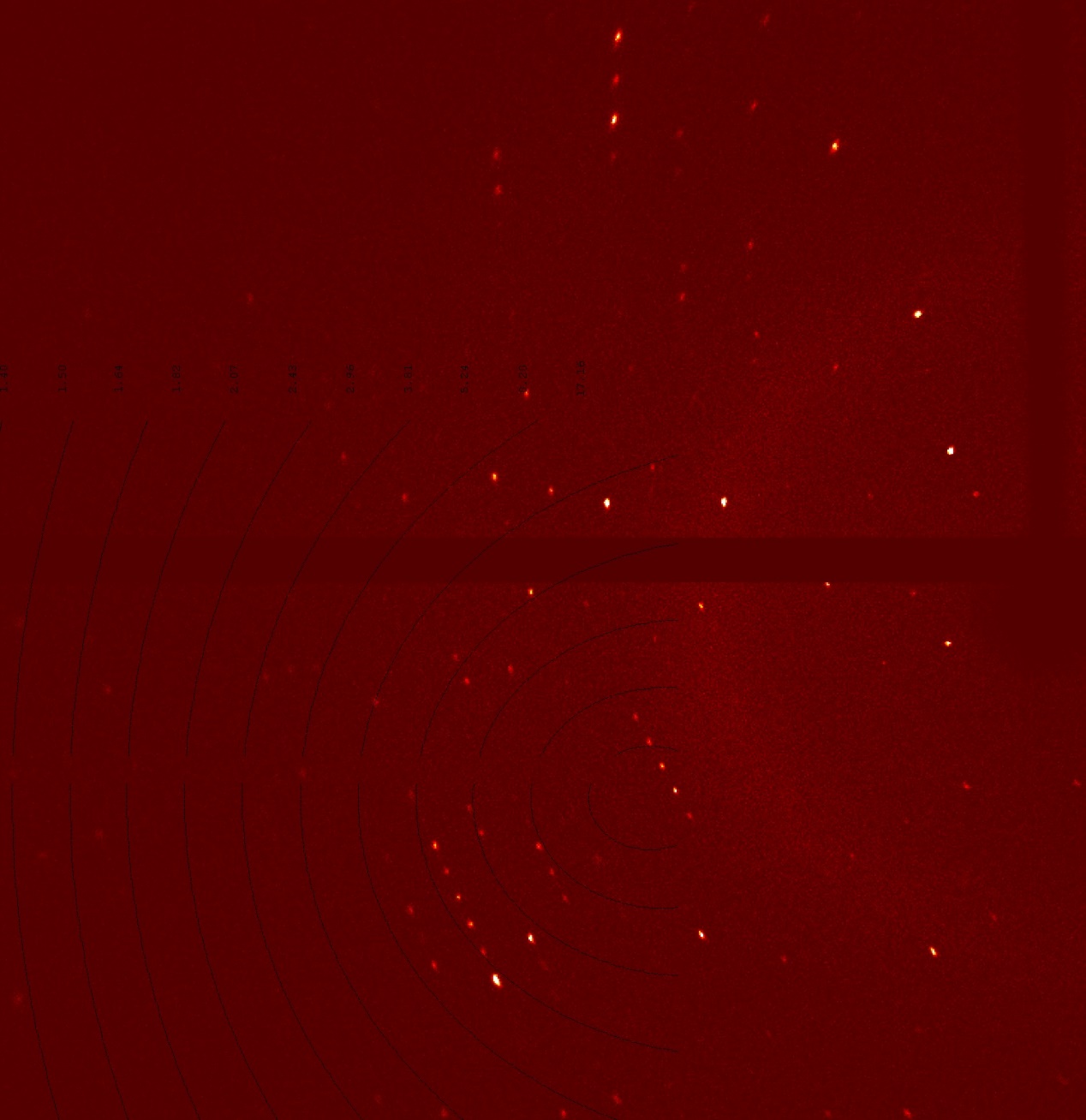


**Figure S3.** Single-crystal X-ray diffraction patterns of (AgCu)_37_.


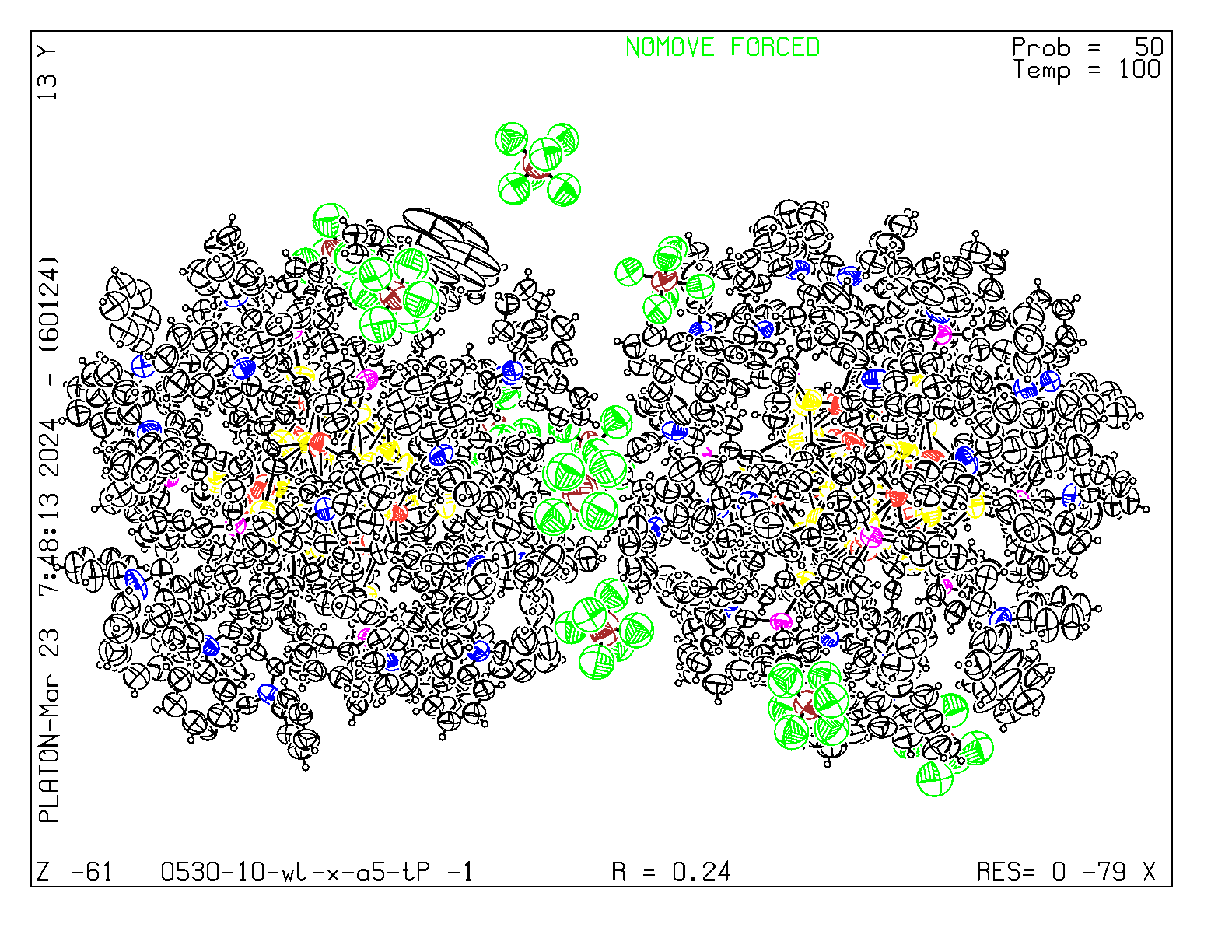


**Figure S4.** The thermal ellipsoids of the ORTEP diagram of the (AgCu)_37_ cluster.


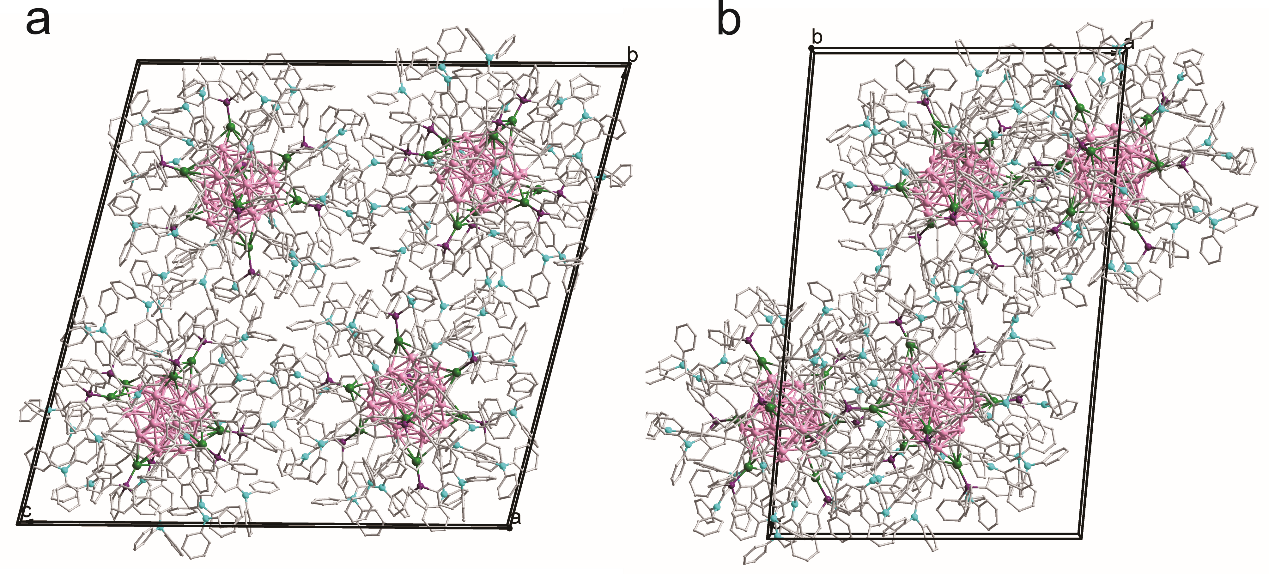


**Figure S5.** The presence of four (AgCu)_37_ cluster molecules in a unit cell.


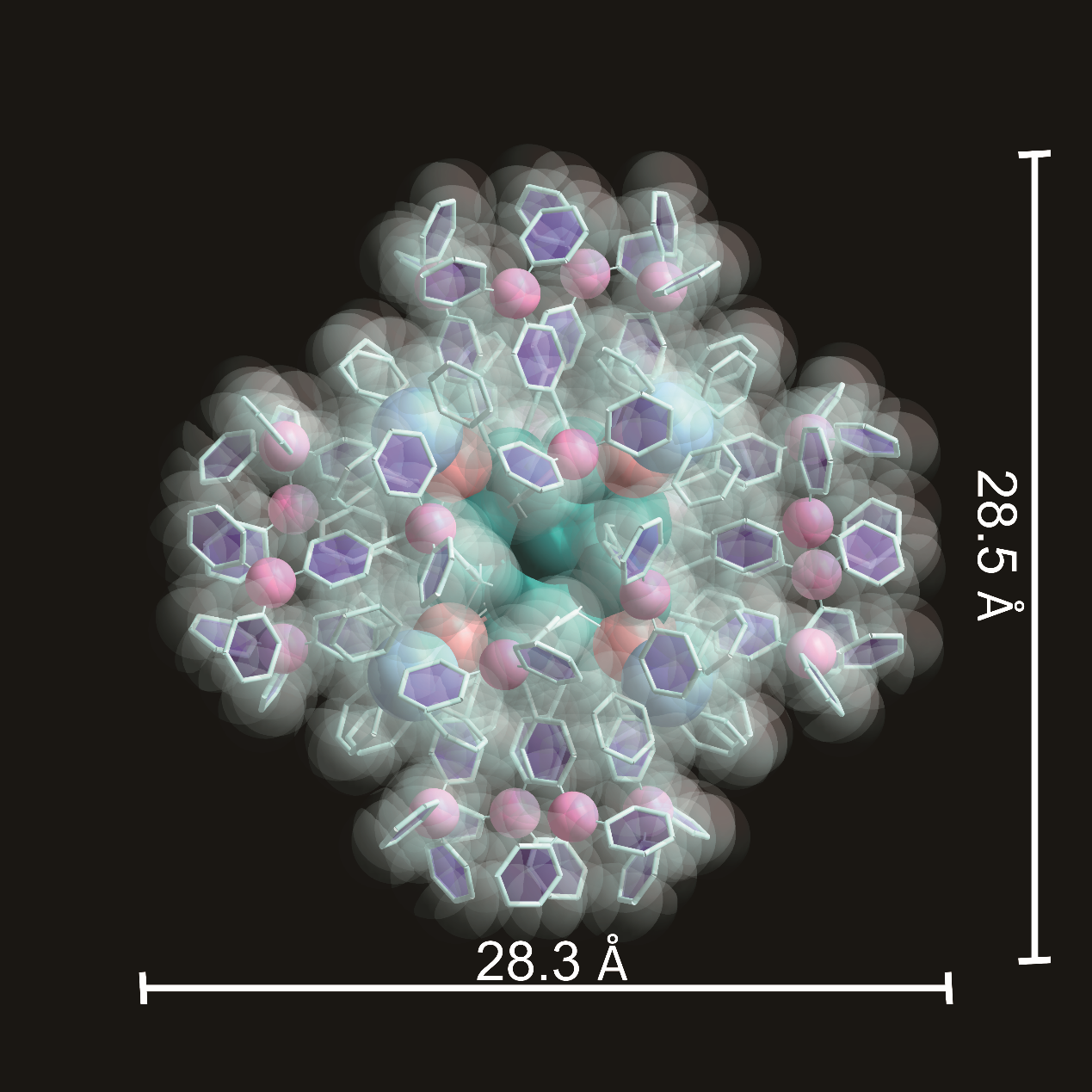


**Figure S6.** Total length of the (AgCu)_37_ cluster.


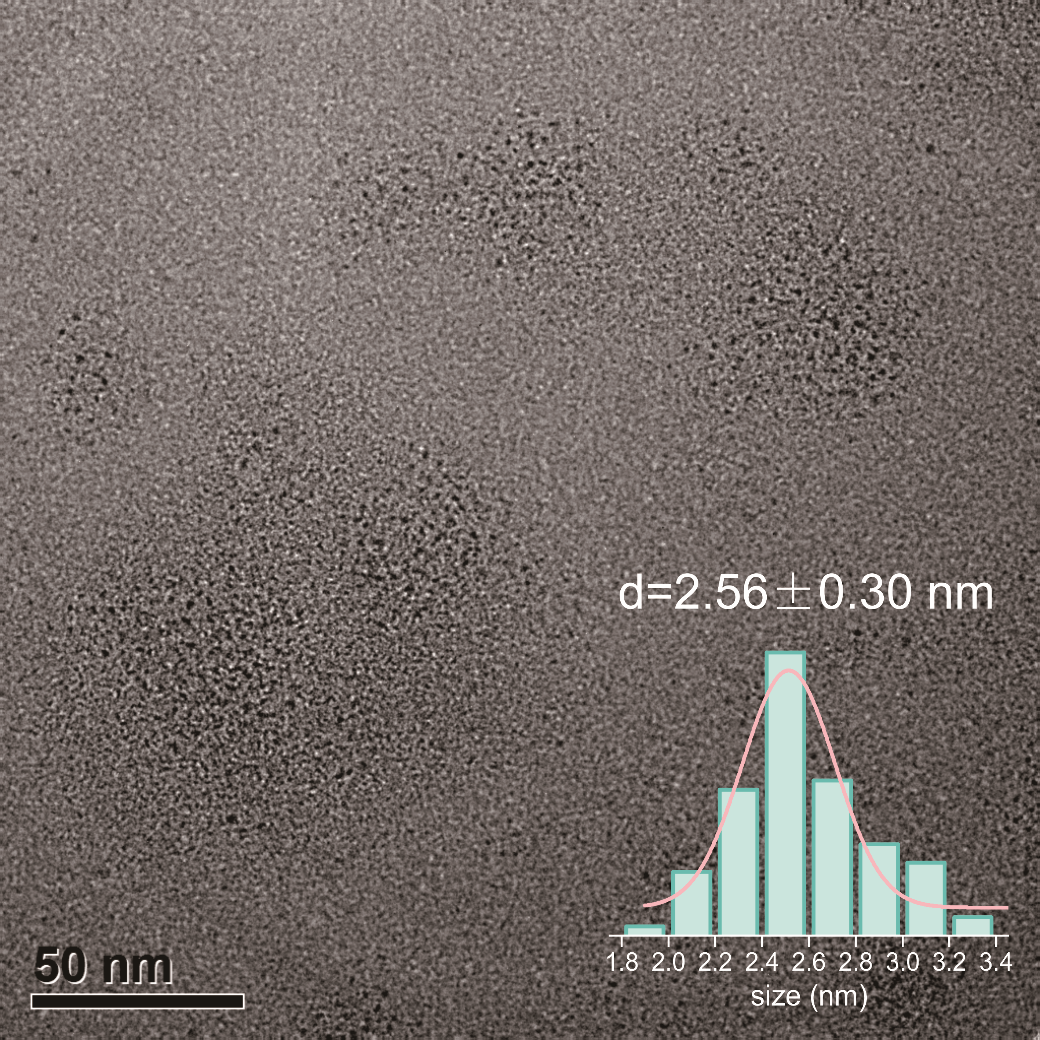


**Figure S7.** TEM characterization of the nanocluster particle size.


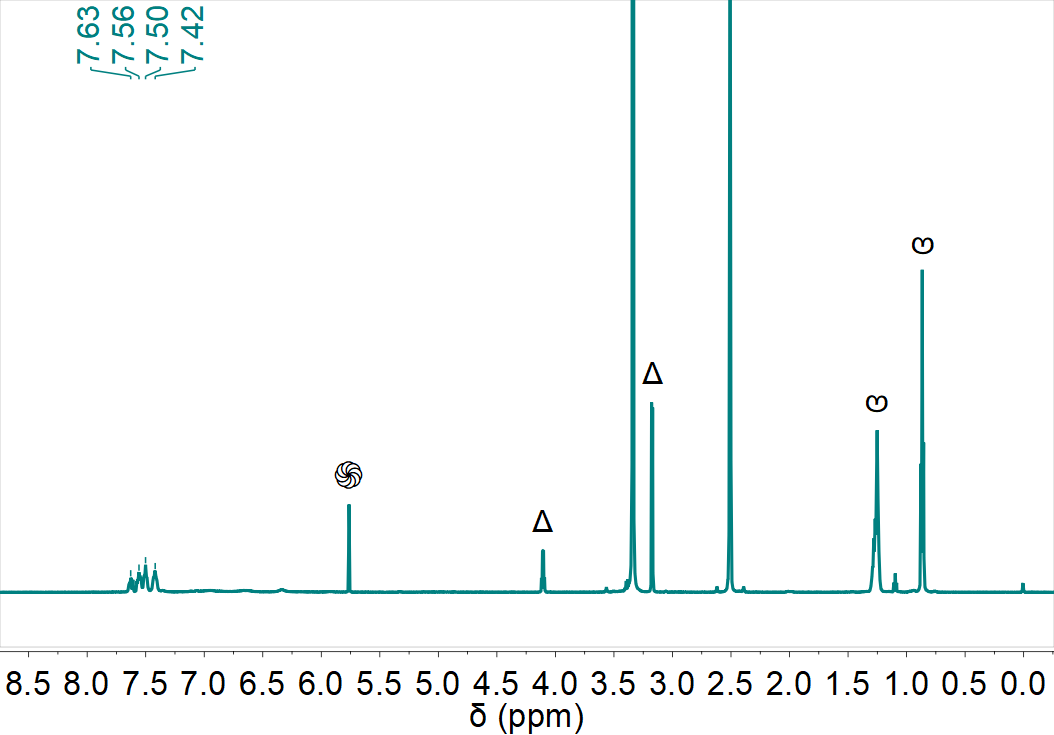


**Figure S8.** ^1^H NMR spectra of (AgCu)_37_ in d_6_-DMSO. δ=0.91 ppm and 1.25 ppm is attributed to n-hexane. δ=3.16 ppm and 4.08 ppm is attributed to methanol. δ=5.76 ppm is attributed to dichloromethane.


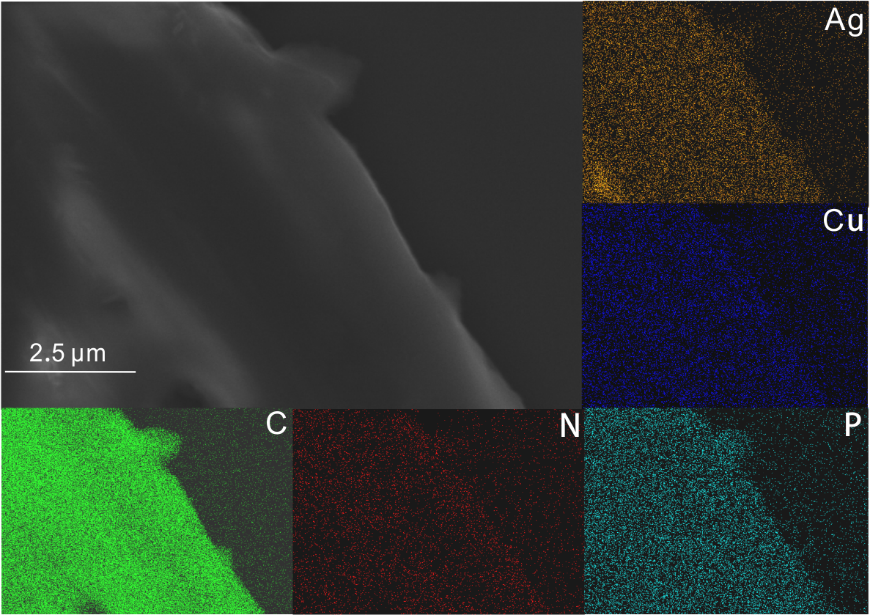


**Figure S9.** The EDS images of the (AgCu)_37_ nanocluster.


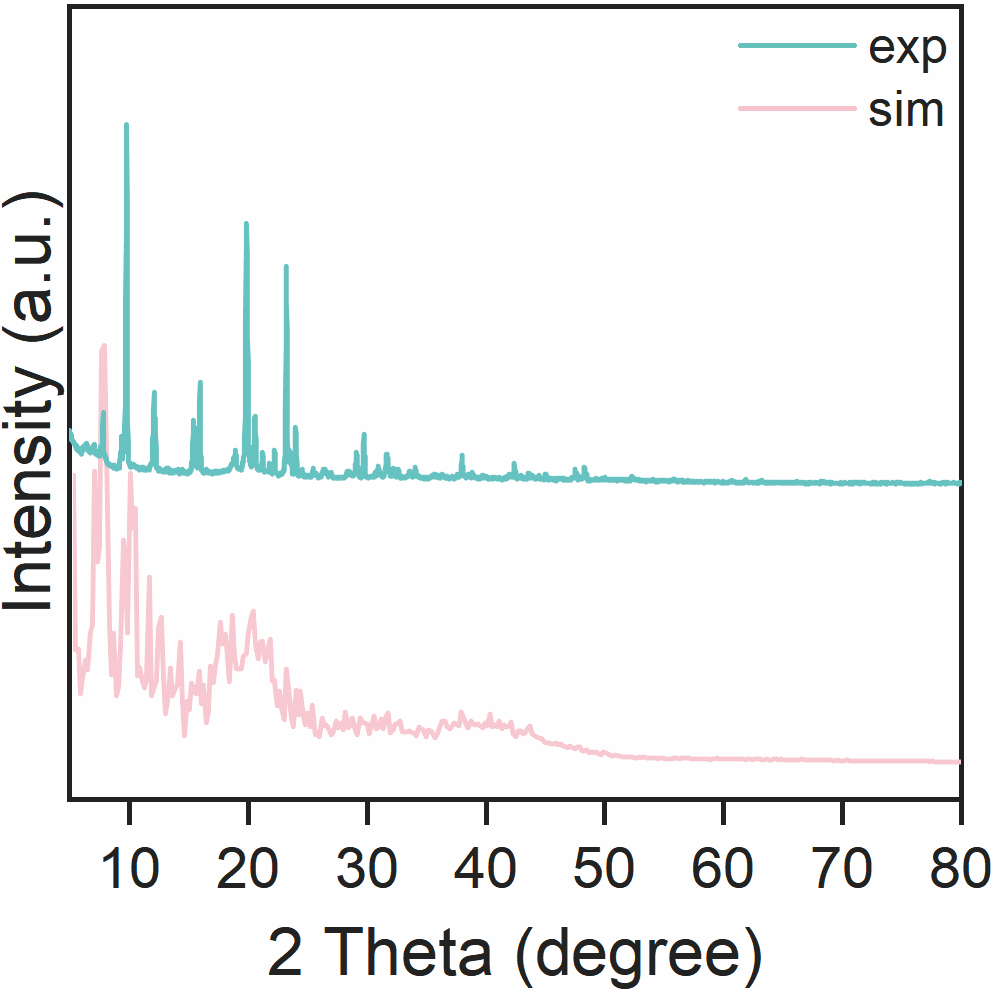


**Figure S10.** PXRD data of the (AgCu)_37_ nanocluster.


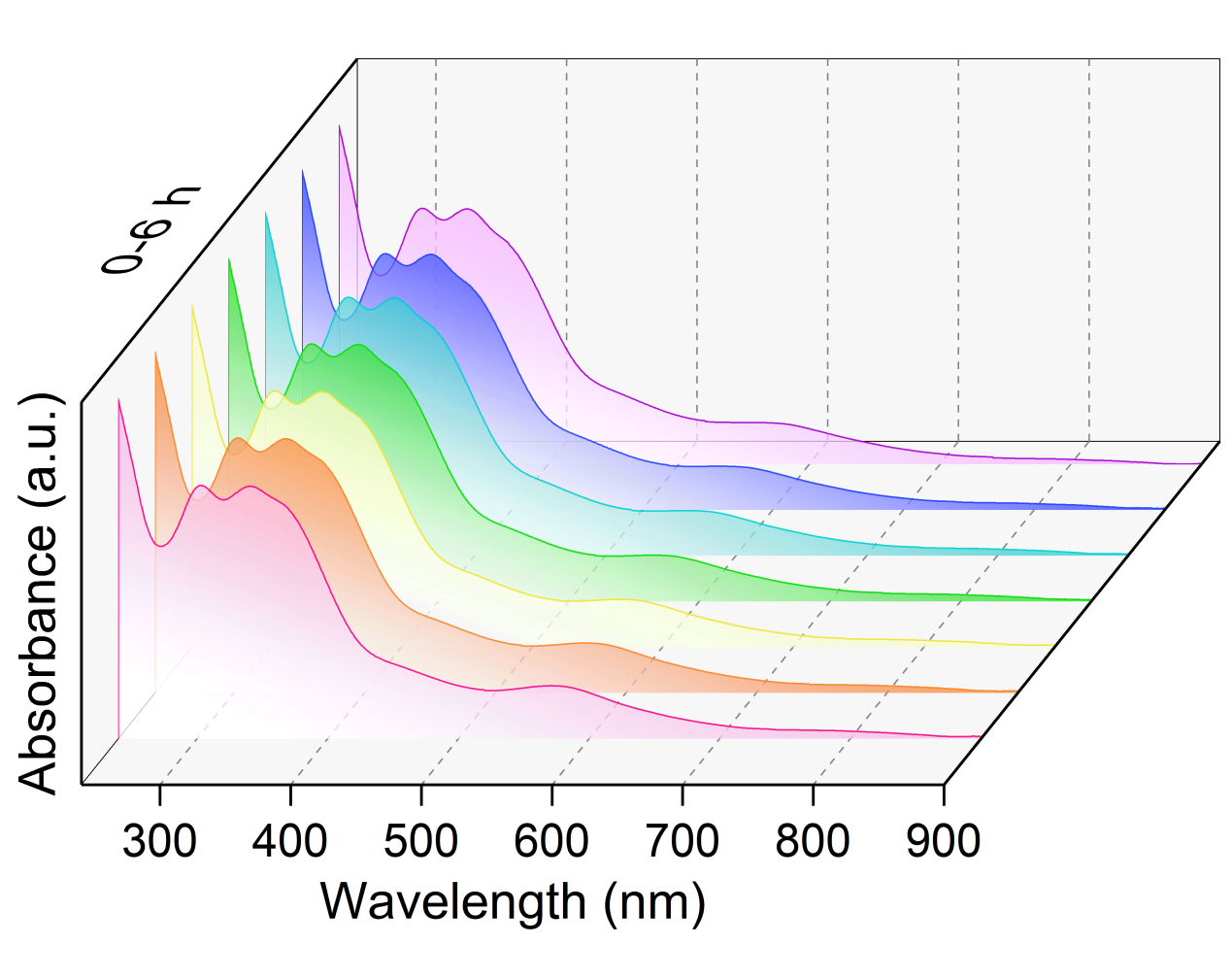


**Figure S11.** Stability of (AgCu)_37_ nanoclusters evaluated by UV-vis spectroscopy. The cluster in dichloromethane was stored in air in the dark.


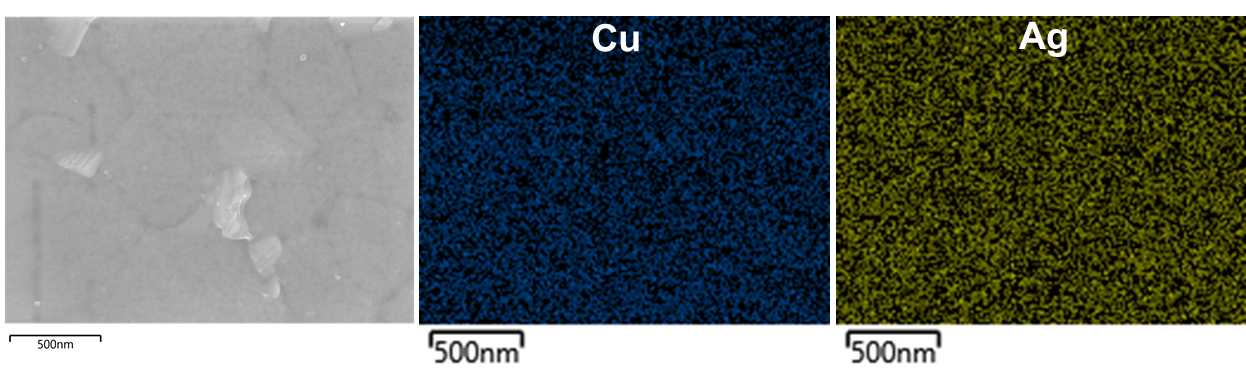


**Figure S12.** Top-view SEM image and EDS mappings of the (AgCu)_37_-modified perovskite film for Cu and Ag elements.


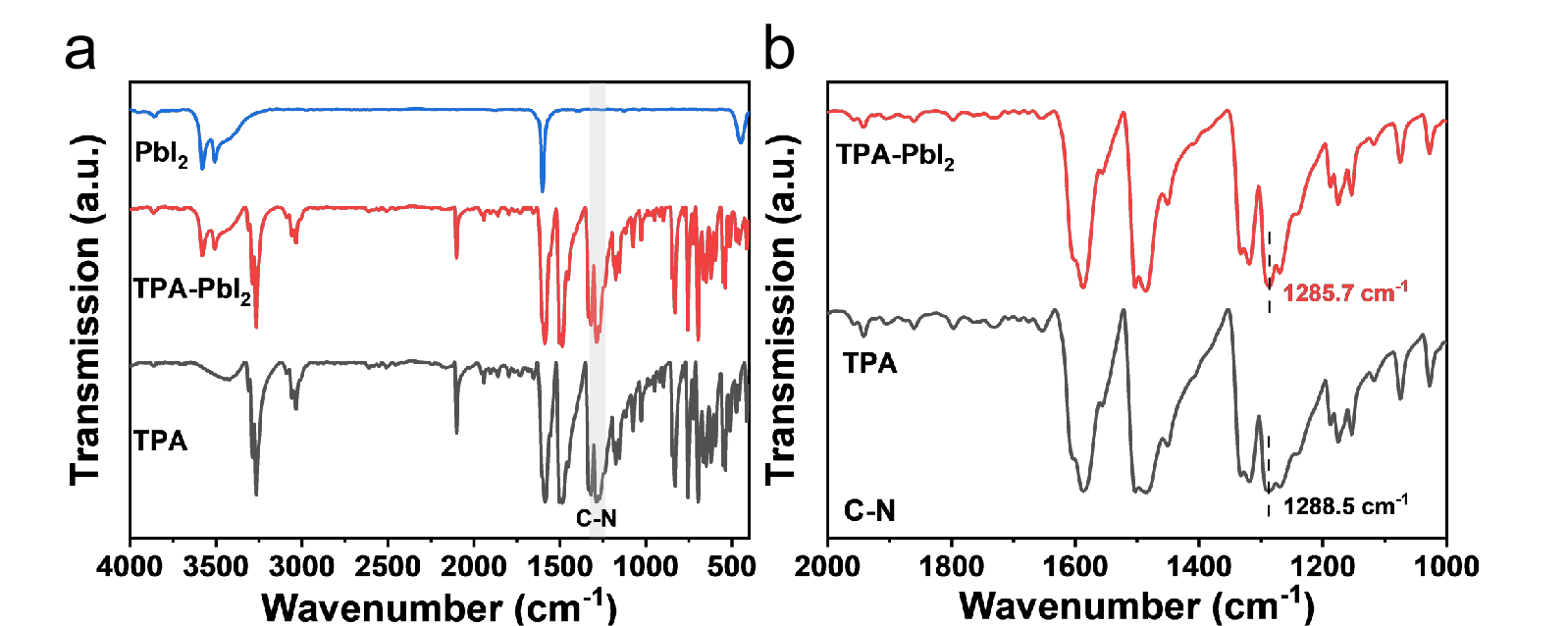


**Figure S13.** Fourier transform infrared spectroscopy (FTIR) for TPA, PbI_2_ and TPA-modified PbI_2_.
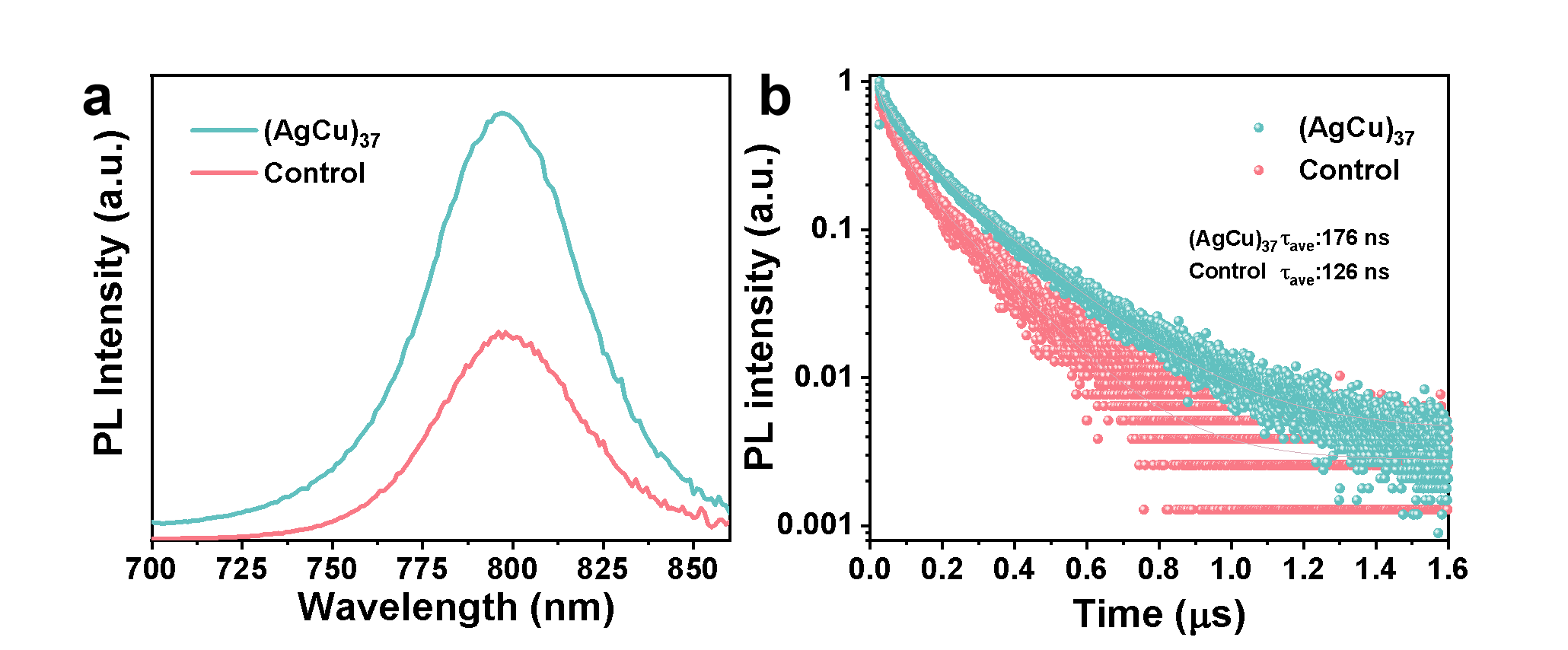


**Figure S14.** (a) PL and (b) TRPL spectra of the (AgCu)_37_-modified and control perovskite films.


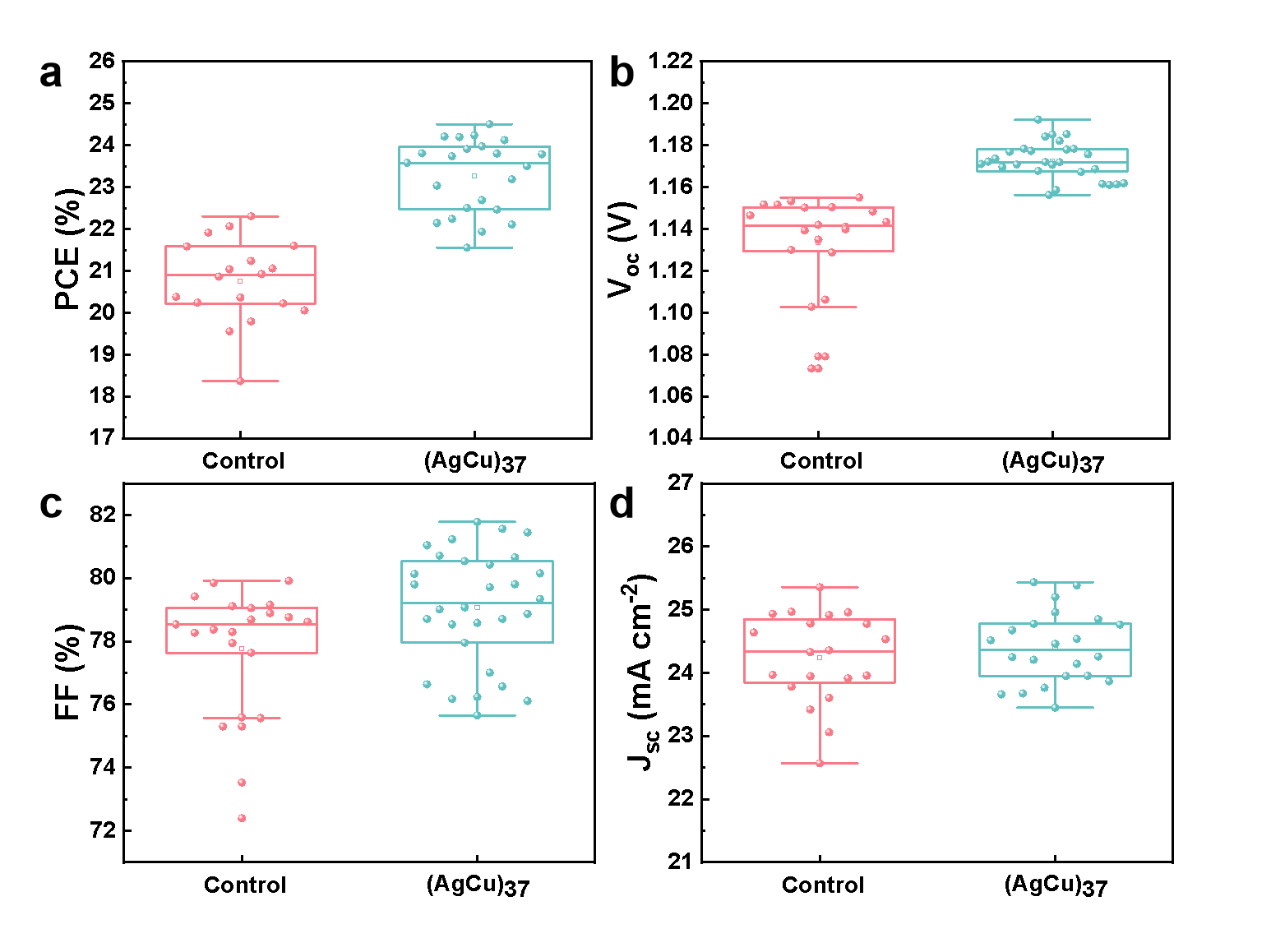


**Figure S15.** (a) PCE, (b) *V*_oc_, (c) FF and (d) current density of Distribution of 20 individual PSCs based on the (AgCu)_37_-modified and control perovskite films.

**Figure S16.** Nyquist plots of the (AgCu)_37_-modified and control PSCs under dark condition with a bias voltage of 0.8 V.

**
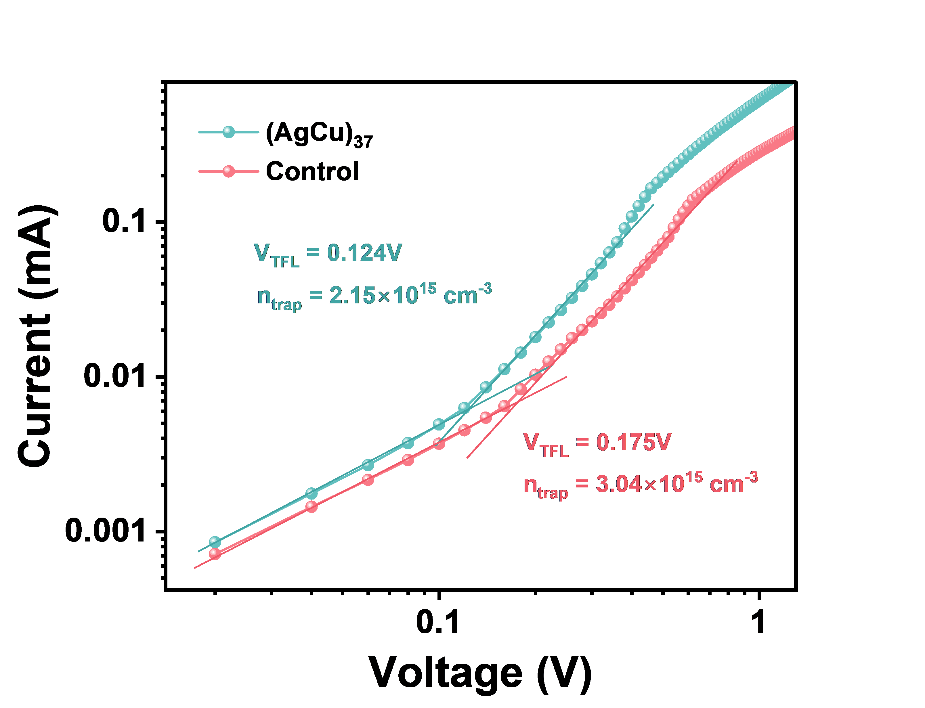
Figure S17.** SCLC plots of hole-only devices (FTO/PEDOT: PSS/perovskite/cluster/Spiro-OMeTAD/Au).


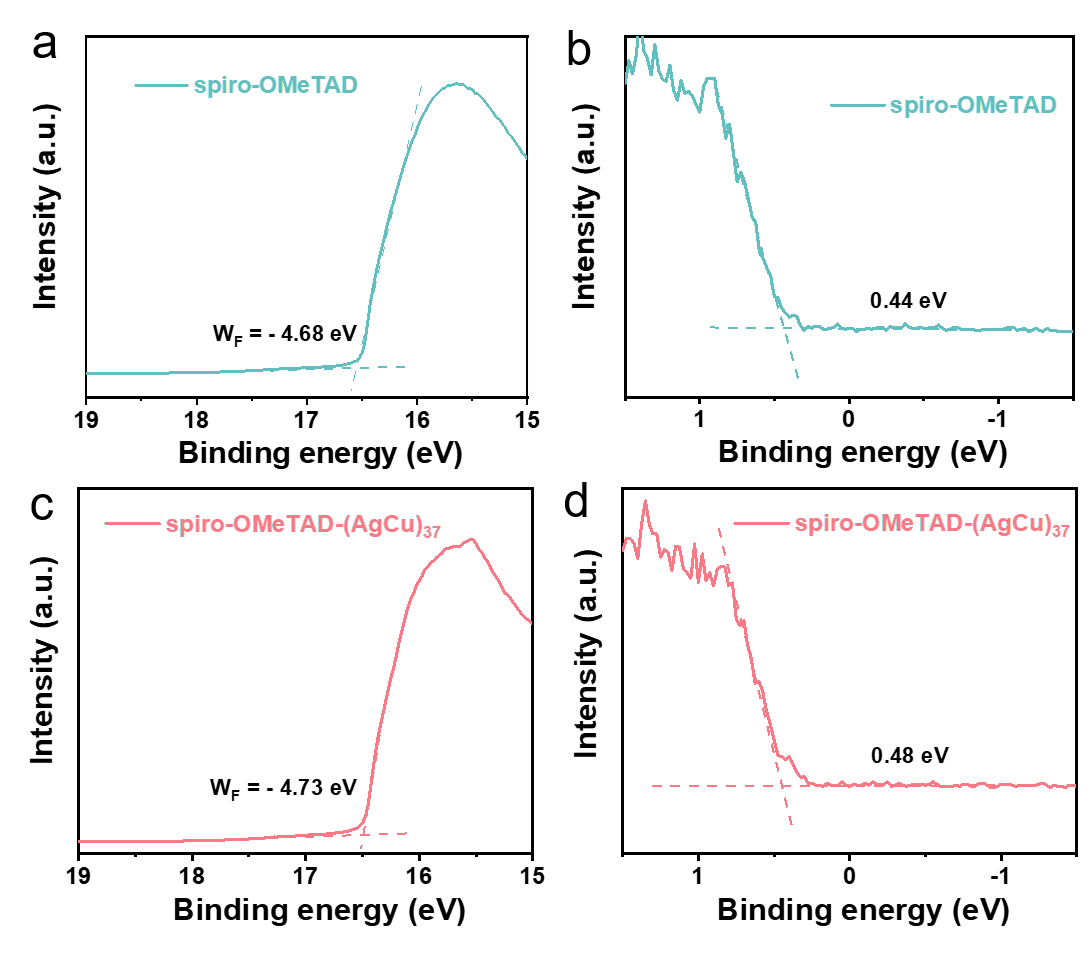


**Figure S18.** Secondary electron cut-off and valence band regions of UPS spectra of the (a, b) control and (c, d) (AgCu)_37_ films.


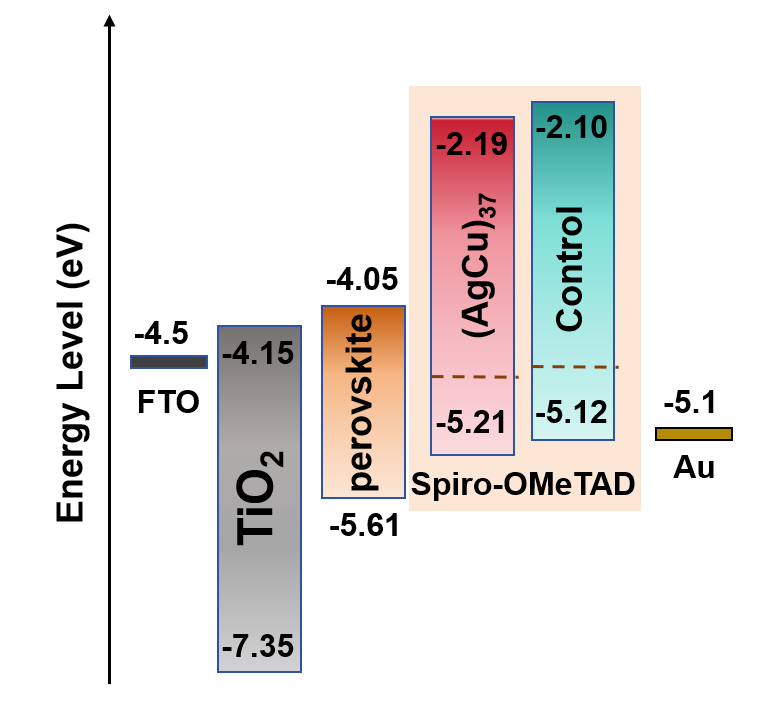


**Figure S19.** Estimated energy level alignment of the control and (AgCu)_37_ PSCs.

**
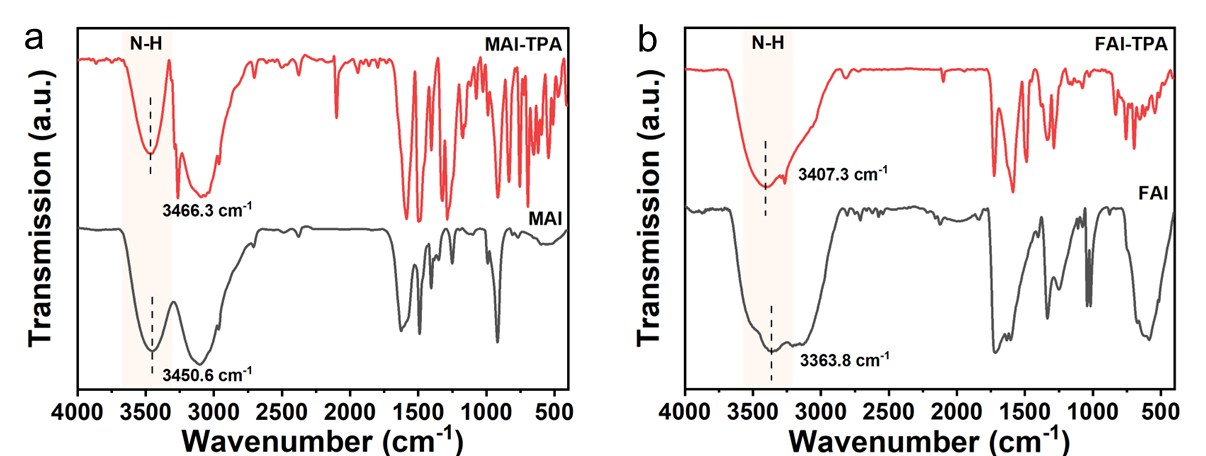
Figure S20.** The possible formation of N- H···N bonds between TPA and organic cations of perovskite, as revealed by FTIR spectroscopy.


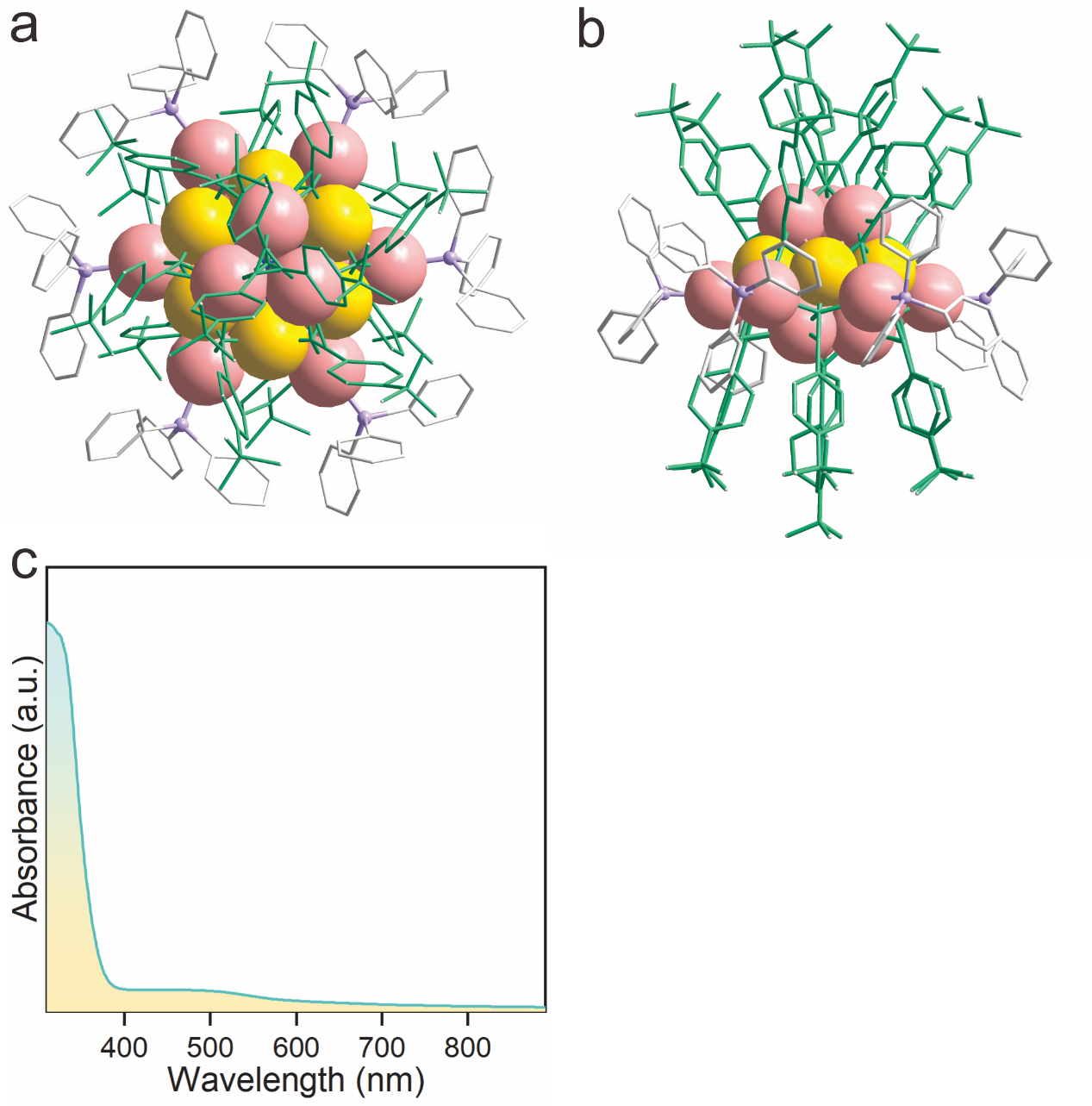


**Figure S21.** Structure and UV-vis spectrum of [Ag_13-x_Cu_6+x_(^t^BuC_6_H_4_C≡C)_14_(PPh_3_)_6_](SbF_6_)_3_.


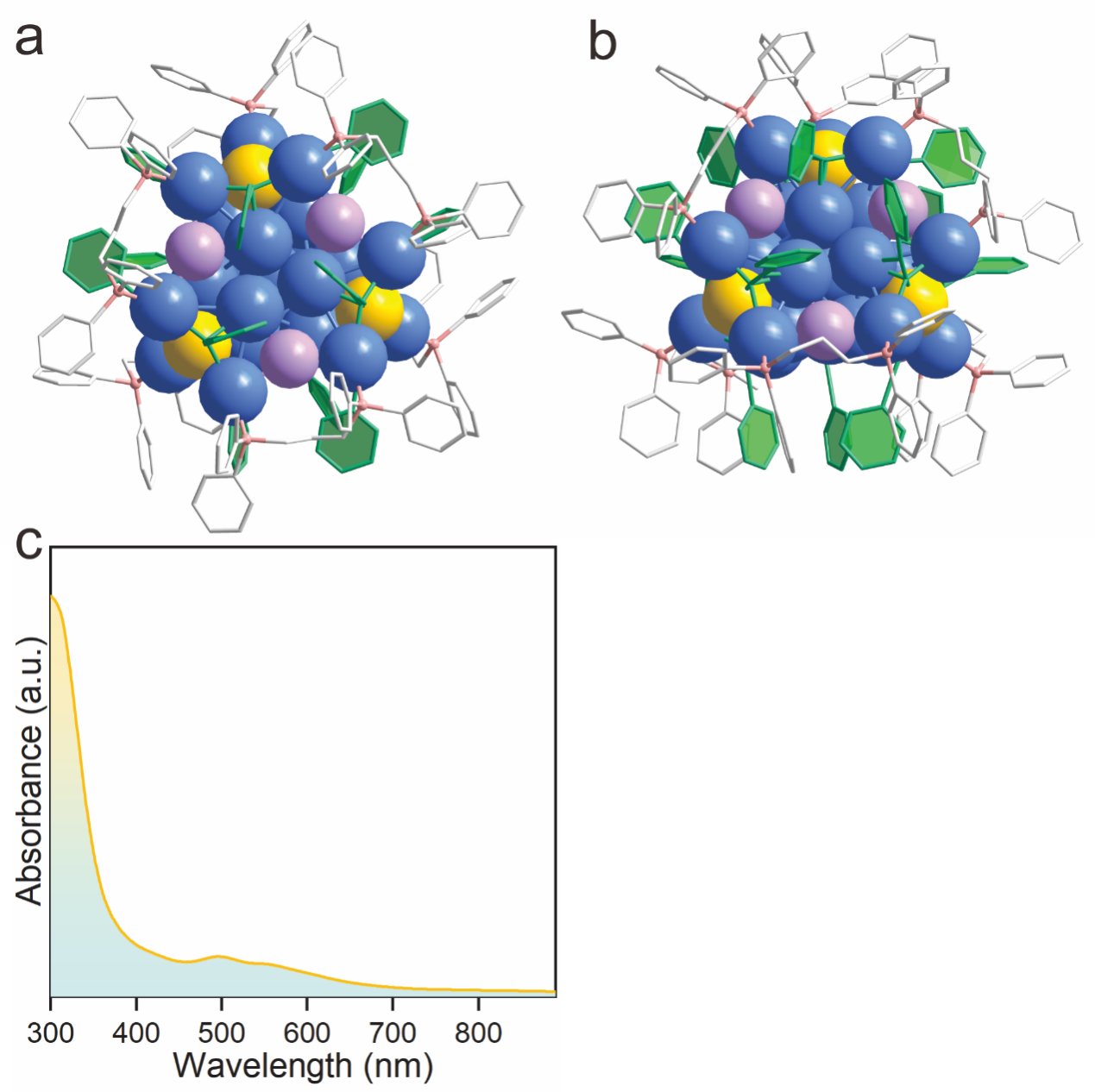


**Figure S22.** Structure and UV-vis spectrum of [Ag_25_Cu_4_(PhC≡C)_12_(PPh_3_)_12_Cl_6_H_8_] (SbF_6_)_3_.


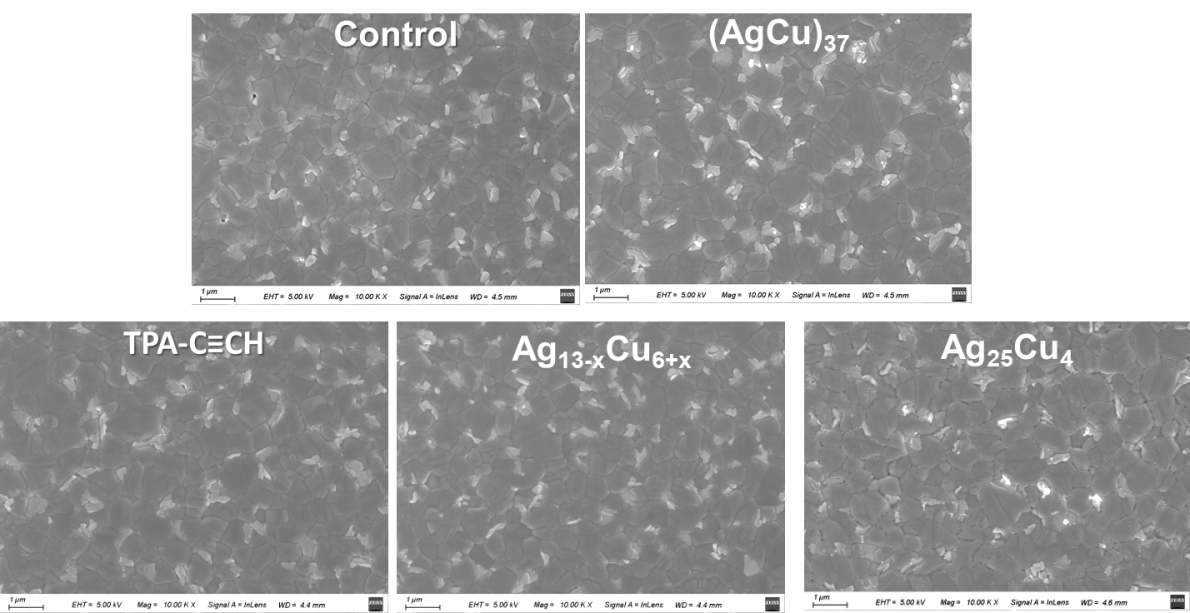


**Figure S23.** Top-view SEM images of the control, (AgCu)_37,_ TPA-C≡CH, Ag_13-x_Cu_6+x_ and Ag_25_Cu_4_ perovskite films.


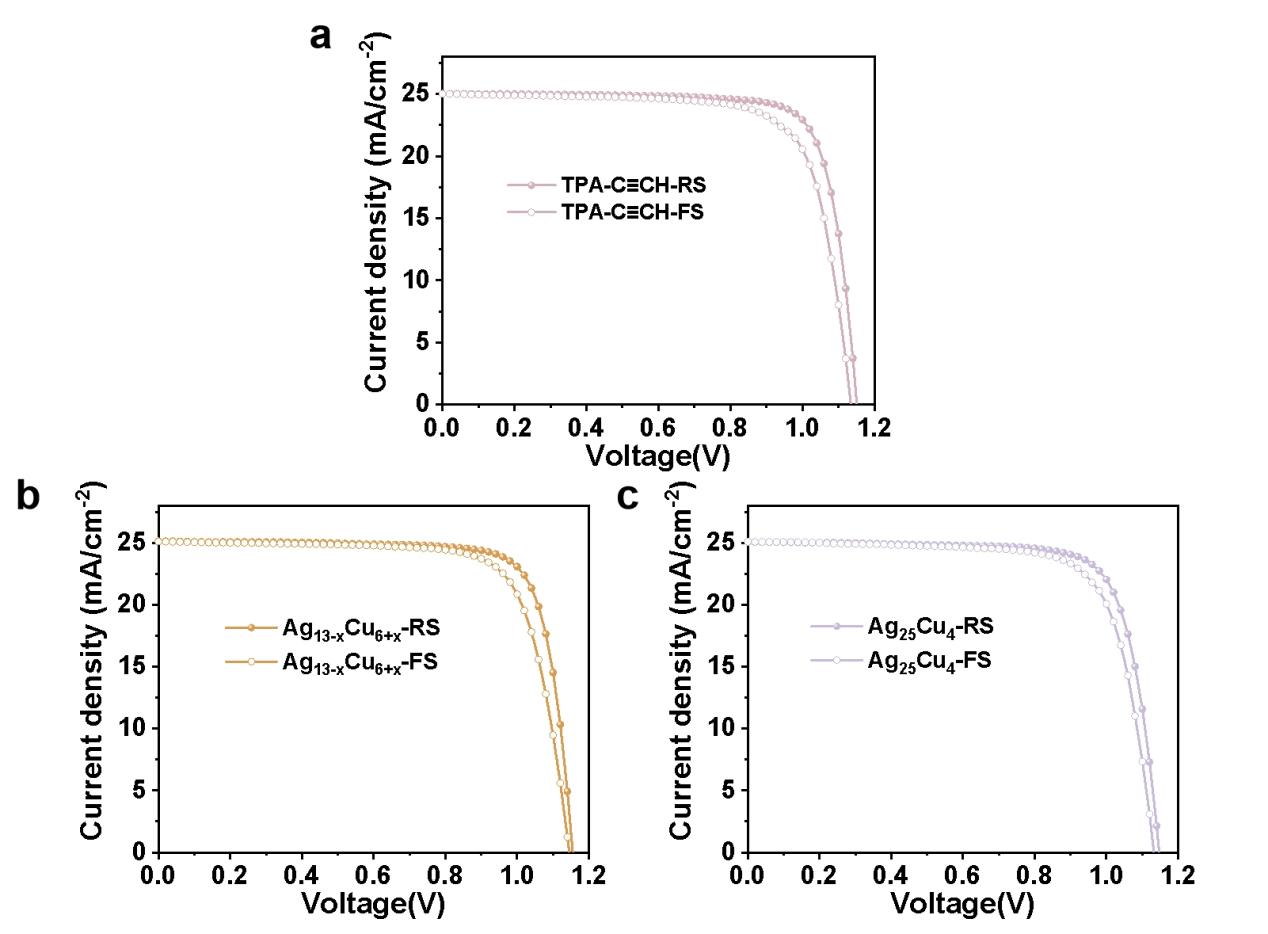


**Figure S24.** *J-V* curves for the best-performance PSCs with TPA-C≡CH, Ag_13-x_Cu_6+x_ and Ag_25_Cu_4_.


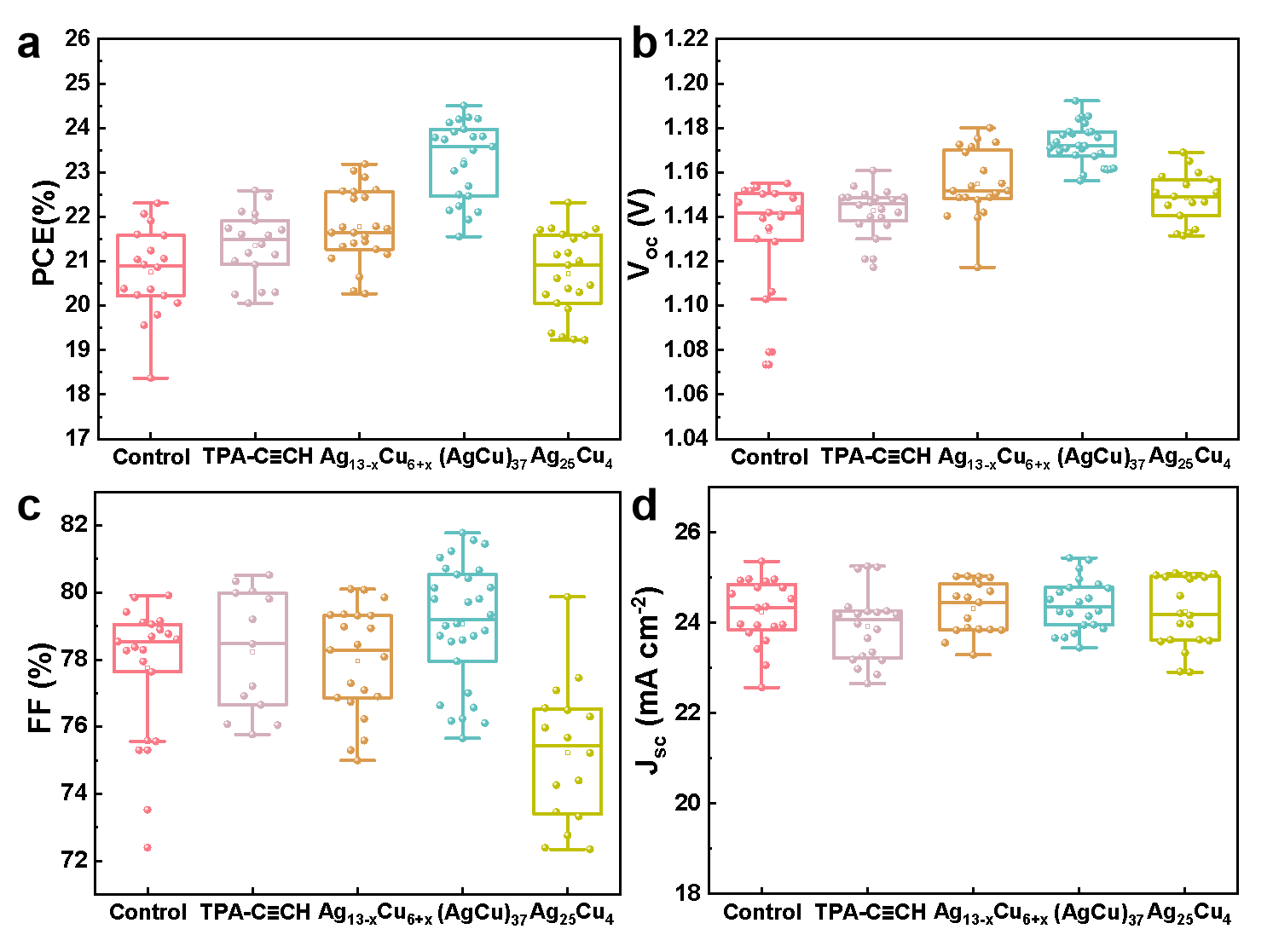


**Figure S25.** (a) PCE, (b) *V*_oc_, (c) FF and (d) current density of Distribution of 20 individual PSCs based on the control, TPA-C≡CH, Ag_13-x_Cu_6+x_, (AgCu)_37_ and Ag_25_Cu_4_ perovskite films.


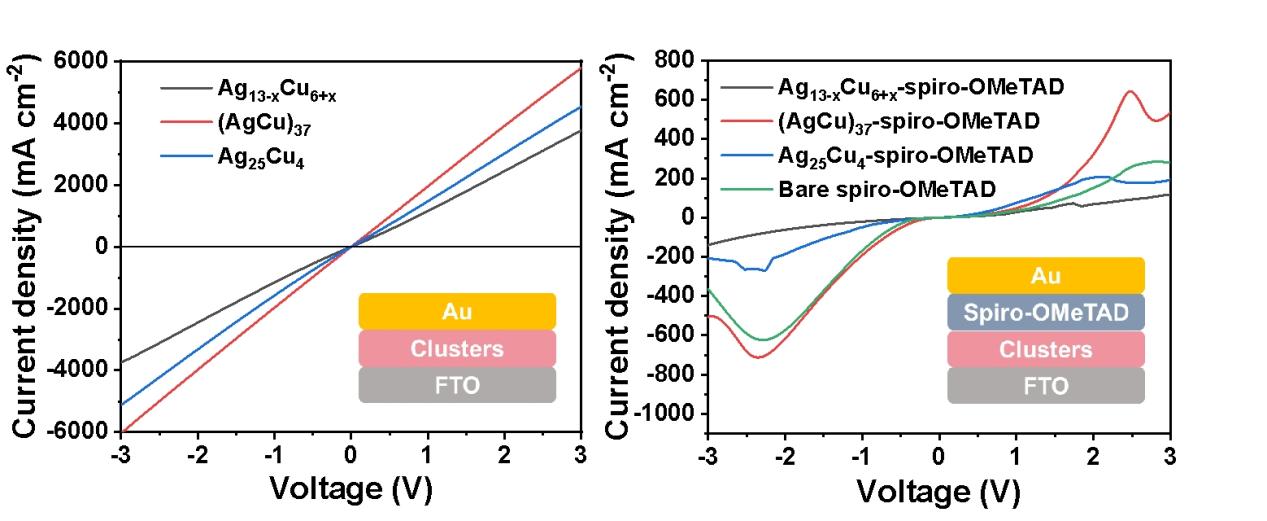
**Figure S26.** Current-voltage curves for the Ag_13-x_Cu_6+x_, (AgCu)_37_ and Ag_25_Cu_4_ devices with the structure of FTO/Clusters/Au and FTO/Clusters/Spiro-OMeTAD/Au obtained at applied voltage -3 to +3 V under dark condition.

**
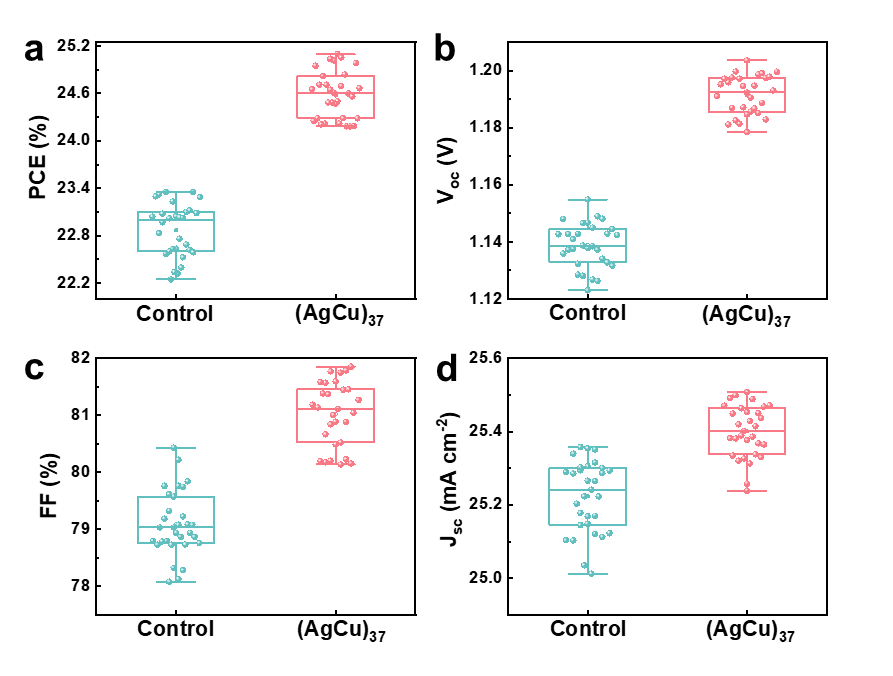
Figure S27.** (a) PCE, (b) *V*_oc_, (c) FF and (d) current density of Distribution of 20 individual PSCs based on the (AgCu)_37_-modified and control FAPbI_3_ perovskite films.

**Table S1.** Photovoltaic parameters of PSCs with control, TPA-C≡CH, Ag_13-x_Cu_6+x_, (AgCu)_37_ and Ag_25_Cu_4_ measured from forward scan and reverse scan.

| Sample | *V_oc_* (V) | *J*_sc_ (mA cm^-2^) | *FF* (%) | PCE (%) |
| --- | --- | --- | --- | --- |
| Control-reverse | 1.143 | 25.06 | 77.87 | 22.31 |
| Control-forward | 1.123 | 24.96 | 75.86 | 21.27 |
| TPA-C≡CH -reverse | 1.151 | 25.01 | 79.69 | 22.93 |
| TPA-C≡CH -forward | 1.134 | 25.00 | 74.36 | 21.09 |
| Ag_13-x_Cu_6+x_ -reverse | 1.155 | 25.13 | 79.48 | 23.07 |
| Ag_13-x_Cu_6+x_ -forward | 1.145 | 25.09 | 75.38 | 21.66 |
| (AgCu)_37_ -reverse | 1.192 | 25.13 | 81.78 | 24.50 |
| (AgCu)_37_ -forward | 1.185 | 25.12 | 80.22 | 23.88 |
| Ag_25_Cu_4_ -reverse | 1.147 | 25.06 | 77.65 | 22.32 |
| Ag_25_Cu_4_ -forward | 1.132 | 25.08 | 74.32 | 21.10 |

**Table S2.** Summary of photovoltaic performance of reported metal cluster-based PSCs in recent years. Note: the theoretical efficiency of FAPbI_3_ is higher than that of the more stable triple-cation FAMACs perovskite.

| **Device structures** | ***V*_oc_ (V)** | ***J*_sc_**  **(mA cm^-2^)** | **FF**  **(%)** | **PCE (%)** | **Ref.** | |
| --- | --- | --- | --- | --- | --- | --- |
| **ITO/SnO_2_/Cs_0.05_(FAMA)_0.95_Pb(I_0.85_Br_0.15_)_3_**:**Au clusters/(BA)_2_PbI_4_/Spiro-OMeTAD/Au** | 1.17 | 23.90 | 73.7% | 20.60% | | *ACS Appl. Energy Mater. 2022, 5, 12663* |
| **FTO/TiO_2_/FAPbI_3_-FGCs/Spiro-OMeTAD/Au** | 1.139 | 25.65 | 82.21 | 24.02% | | *Adv. Mater. 2021, 36, 2101590* |
| **FTO/TiO_2_/FAPbI_3_-ABSA/Spiro-OMeTAD/Au** | 1.175 | 25.46 | 83.55 | 25.00% | | *Adv. Mater. 2024, 36, 2310651.* |
| **FTO/TiO_2_/Cs_0.05_FA_0.85_MA_0.1_PbI_2.9_Br_0.1_/**  **cluster/Spiro-OMeTAD/Ag** | **1.19** | **25.13** | **81.78** | **24.50** | | ***This work*** |
| **FTO/TiO_2_/ FAPbI_3_/**  **cluster/Spiro-OMeTAD/Ag** | **1.204** | **25.48** | **81.86** | **25.10** | | ***This work*** |

**References**

[1] a) Y. Gao, X. Sun, X. Tang, Z. Xie, G. Tian, Z. Nan, H. Yang, H. Shen, *Dalton Trans.* **2023**, *52*, 52; b) H. Shen, Y. Han, Q. Wu, J. Peng, B. K. Teo, N. F. Zheng, *Small Methods* **2021**, *5*, 2000603.

[2] G. M. Sheldrick, *Acta Cryst. A* **2015**, *71*, 3.

[3] G. M. Sheldrick, *Acta Cryst. A* **2008**, *64*, 112.

[4] C. B. Hubschle, G. M. Sheldrick, B. Dittrich, *J. Appl. Cryst.* **2011**, *44*, 1281.

[5] O. V. Dolomanov, L. J. Bourhis, R. J. Gildea, J. A. K. Howard, H. Puschmann, *J. Appl. Cryst.* **2009**, *42*, 339.
